# Breaking barriers: nanosystems to overcome solubility and permeability challenges of second-generation tyrosine kinase inhibitors

**DOI:** 10.3762/bjnano.17.73

**Published:** 2026-08-10

**Authors:** Bhagya Shree, Abhishek Sharma, Manish Kumar, Ruchi Chawla, Brahmeshwar Mishra

**Affiliations:** 1 Department of Pharmaceutical Engineering and Technology, Indian Institute of Technology (BHU), Varanasi, Uttar Pradesh, 221005, India; 2 Scientist and Deputy Manager, Formulation and Development, Research and Development, Cadila Pharmaceuticals Limited, Survey No. 1389, Trasad Road, Dholka, Ahmedabad, Gujarat, 382225, India

**Keywords:** BCS class-IV, bioavailability enhancement, biopharmaceutical limitation, bosutinib, dasatinib, drug delivery system, nanosystems, nilotinib, quality by design, second-generation TKI

## Abstract

Second-generation tyrosine kinase inhibitors (TKIs) – bosutinib, nilotinib, and dasatinib – demonstrate therapeutic efficacy across various chronic malignancies, including chronic myelogenous leukaemia, acute lymphoblastic leukaemia, breast cancer, lung cancer, pancreatic cancer, and hepatocellular cancer. However, in the Biopharmaceutics Classification System, they are classified as class-II and class-IV drugs, exhibiting poor, pH-dependent solubility and low permeability, leading to poor and unpredictable bioavailability and variable plasma concentrations, compromising their clinical efficacy. Even though the oral route is the most widely accepted mode of administration, systematic analysis of the formulation database revealed that the conventional formulations encounter biopharmaceutical barriers limiting the therapeutic performance of drugs. Hence, this review examines nanotechnology-driven solutions that may improve the second-generation TKIs’ biopharmaceutical properties for better therapeutic performance by enhancing their pharmacokinetic properties. Various nanosystems, such as solid lipid nanoparticles, nanostructured lipid carriers, polymeric micelles, polymeric nanoparticles, nanocrystals, self-nanoemulsifying drug delivery systems, and nanosuspensions, have been developed to enhance solubility, permeability, and bioavailability. The nanoformulations achieved variable improvements in solubility and bioavailability (1.5- to 38-fold) compared with free drugs, depending on surface characteristics, carrier composition, and drug properties. This was possible through tailored particle size, drug loading, and precise drug release kinetics via various mechanisms. This review consolidates the evidence from peer researchers on positioning nanoformulations as a transformative tool to improve the efficacy of second-generation TKIs. Critical evaluation was performed based on solubility/permeability enhancement potential, safety profile, toxicological burden, long-term stability, scalability, and feasibility for clinical transition.

## Introduction

**Figure 1:**
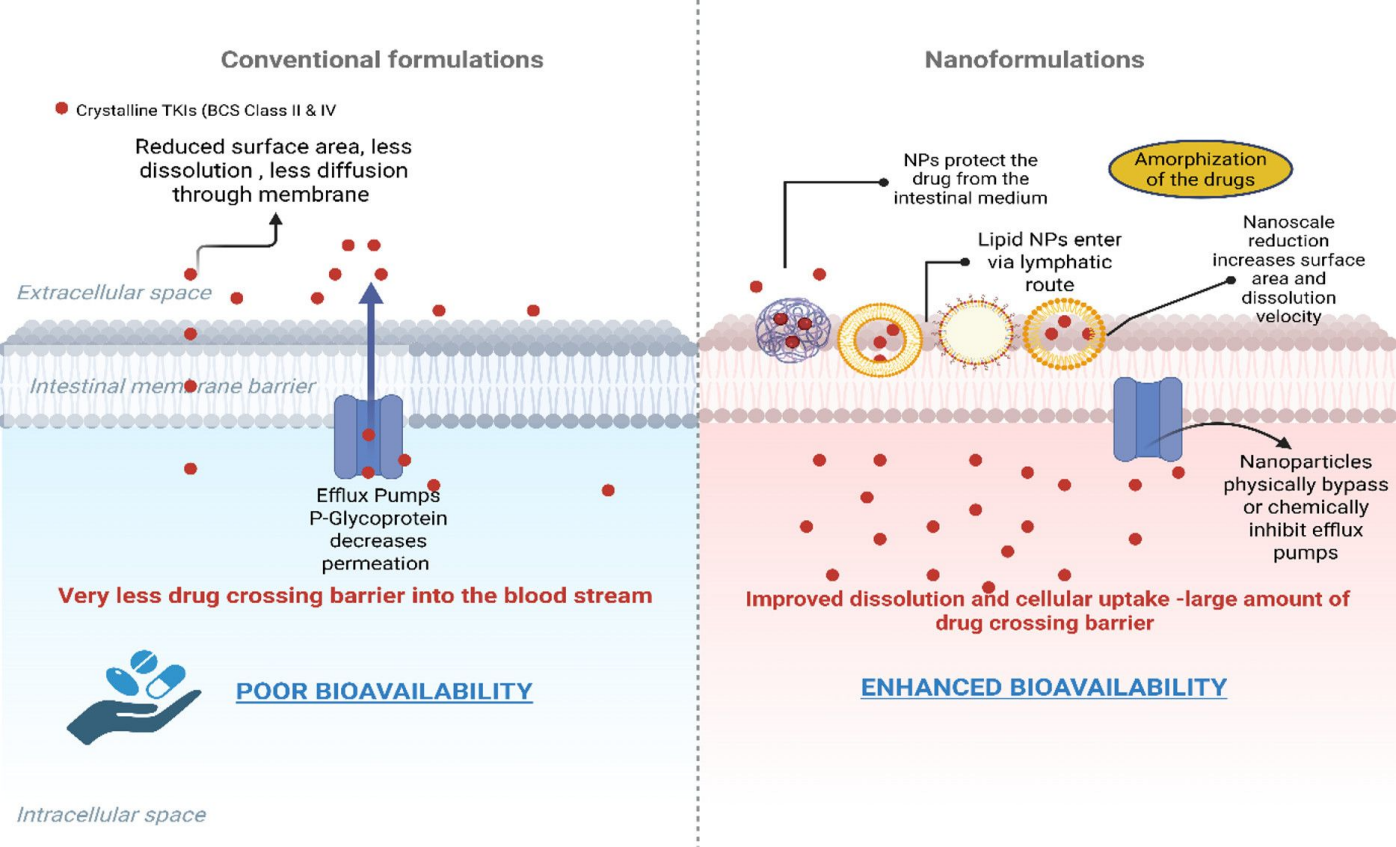
Mechanistic comparison of drug absorption: conventional versus nanoformulation approaches. Figure 1 was created in BioRender. Mishra, B. (2026) https://BioRender.com/pjdijin. This content is not subject to CC BY 4.0.

Tyrosine kinases have emerged as important targets in cancer chemotherapy. These enzymes transfer a phosphate group (primarily the terminal phosphate of adenosine triphosphate (ATP)) to the protein, thereby activating its function. Inhibiting this enzyme’s triggered signalling pathway inactivates various proteins and downregulates their activity, thereby suppressing tumour cell growth, progression, and recurrence. Common protein families of tyrosine residues that act as substrates for tyrosine kinase inhibitors (TKIs) include anaplastic lymphoma kinase, breakpoint cluster region–Abelson (BCR-ABL), Bruton’s tyrosine kinase, mesenchymal epithelial transition factor, epidermal growth factor receptor, Janus kinase, platelet-derived growth factor receptor, receptor tyrosine kinase, Sarcoma (Src), and vascular endothelial growth factor receptor [1]. The three main second-generation TKIs are bosutinib (BTB), nilotinib (NTB), and dasatinib (DTB), which target the Src family protein and the BCR-ABL protein to treat various types of cancers by overcoming the drug resistance and potency issues posed by first-generation TKIs including imatinib, sunitinib, and sorafenib [2]. DTB, NTB, and BTB are approved for patients with chronic phase Philadelphia chromosome-positive chronic myelogenous leukaemia (Ph+ CML), newly diagnosed or resistant or intolerant to prior therapy. Additionally, BTB is also indicated in patients with accelerated phase or blast phase Ph+ CML. At the same time, DTB is indicated for Ph+ve acute lymphoblastic leukaemia. The associated benefits of these three over imatinib are 30-fold potency of NTB [3], 325-fold potency of DTB against BCR-ABL kinase activity in vitro [4], and minimal off-target non-specific response of BTB [5]. BTB, NTB, and DTB share common structural features, including a nitrogen-heterocyclic core and an extensive aromatic ring system, which are required for high therapeutic activity but also contribute to formulation challenges. The various physicochemical properties of the drugs are summarized in Table 1 [6]. The drugs BTB and NTB do not follow the “Lipinski rule of five” (molecular weight ≤ 500 Daltons, log *P* ≤ 5, H-bond donors ≤ 5, H-bond acceptors ≤ 10, molar refractivity = 40–130), which denotes how well the drug will be absorbed into the bloodstream; instead, they have molecular weights of more than 500 Da and a log-*P* value greater than 5 (DTB has poor bioavailability due to all other factors) [7]. All three drugs exhibit pH-dependent solubility, affecting their dissolution in the physiological pH range. A brief overview of the pharmacokinetics of the drugs is also summarized in Table 2. These TKIs belong to the Biopharmaceutical Classification System (BCS) classes II (DTB) and IV (BTB and NTB), posing challenges in formulation development and drug absorption [8]. Due to biopharmaceutical limitations, including poor aqueous solubility and low intestinal permeability, these drugs must be administered at high doses to achieve the desired therapeutic effect [9]. For example, the recommended daily doses are 600–800 mg for NTB (Tasigna^®^, AMN107), 400–500 mg for BTB (Bosulif^®^, SKI-606) and 100–140 mg for DTB (Sprycel^®^, BMS-354825). Such high doses are associated with a high risk of dose-related adverse effects and a higher overall cost of therapy [10]. All these factors can be addressed by novel nanotechnology-enabled formulation strategies that improve solubility at physiological pH, enhance drug absorption and permeation, and protect against an adverse microenvironment, thereby improving drug bioavailability and reducing the dose requirement. The fundamental differences in drug transport and absorption between conventional formulations and nanotechnology-enabled systems are illustrated in Figure 1. The second-generation TKIs are substrates for the P-glycoprotein (P-gp)/breast cancer resistance protein (BCRP) efflux transporter at the brush-border and basolateral membranes of the enterocytes, further limiting absorption, as these counter transport the absorbed drug back into the lumen [11]. However, when loaded into nanocarriers, pre-clinical models suggest that various mechanisms contribute to transporter inhibition to improve intracellular accumulation; for instance, the excipient ᴅ-α-tocopheryl polyethene glycol succinate (TPGS) can inhibit the efflux transporter (P-gp ATPase activity) [12]. Other mechanisms include lymphatic bypass, altered membrane fluidity (solid lipid nanoparticles (SLNs), nanostructured lipid carriers (NLCs), and liposomes), receptor-mediated endocytosis (cationic nanoparticles and receptor-targeted nanoparticles) [13] and ATP depletion by pluronic-based nanovehicles, leading to mitochondrial dysfunction [14]. Hence, conventional approaches to improve solubility/permeability (micronization, co-solvents, and use of buffers to maintain pH) provide only marginal improvement for BCS class-II/IV drugs at therapeutically relevant doses. The use of nanotechnology may offer unique advantages (although they come with their challenges). A few advantages are (i) increased surface area-to-volume ratio, enhancing the dissolution profile of the drugs; (ii) by-passing first pass metabolism via lymphatic uptake; (iii) mucoadhesive properties that lead to prolonged residence time of the drug in the gastrointestinal tract (GIT); and (iv) protection of the drugs after encapsulation into carriers, which evades recognition by P-gp [15].

**Table 1:** Physicochemical properties of drugs.

Property	Dasatinib^a^	Nilotinib^b^	Bosutinib^c^	Ref.

chemical structure	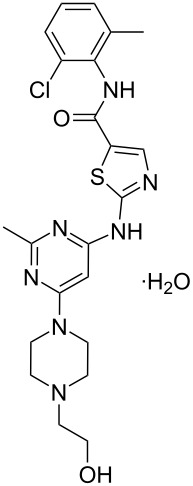	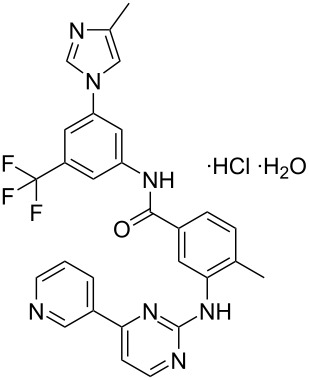	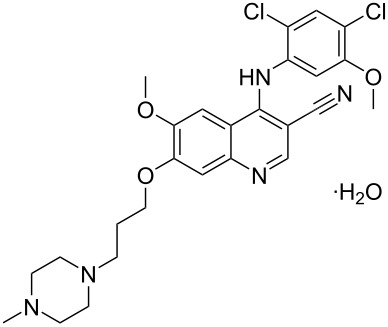	

molecular weight (Da)	488.01	529.51	530.44	[17]
log *P*	3.1	5.3	5.9	
H-bond donors	3	2	1	
H-bond acceptors	8	7	8	
p*K*_a_	6.8, 3.1, 10.8	2.1, 5.4	7.1	
solubility (mg/mL)	≈18 at pH 2.6, <0.001 at pH 7.0	insoluble at pH ≥ 4.5	11.03 at pH 1.0; 0.02 at pH 6.8	[6]
permeability (× 10^−6^ cm/s)	10.2 at 50 µm (high)	moderate	2.08–2.96 (low)	

^a^IUPAC name [16]: (*N*-(2-chloro-6-methylphenyl)-2-[[6-[4-(2-hydroxyethyl)piperazin-1-yl]-2-methylpyrimidin-4-yl]amino]-1,3-thiazole-5-carboxamide monohydrate); ^b^IUPAC Name [16]: (4-methyl-*N*-[3-(4-methyl-1*H*-imidazol-1-yl)-5-(trifluoromethyl)phenyl]-3-[[4-(pyridin-3-yl)pyrimidin-2-yl]amino]benzamide monohydrochloride monohydrate); ^c^IUPAC Name [16]: (4-[(2,4-dichloro-5-methoxyphenyl)amino]-6-methoxy-7-[3-(4-methylpiperazin-1-yl)propoxy]quinoline-3-carbonitrile monohydrate).

**Table 2:** Pharmacokinetic parameters of drugs.

Parameter	Dasatinib	Nilotinib	Bosutinib	References

half-life (h)	3–5	15–24	32.4–41.2	[18]
oral bioavailability (%)	14–34	32	34	[6]
protein binding (%)	96	99	94	[18]
volume of distribution (L/kg)	35	–	86	
clearance (L/h)	363.8	–	189	
metabolized by	CYP3A4	CYP3A4, CYP2C8	CYP3A4	
efflux transporters	P-gp and BCRP	P-gp and BCRP	P-gp, BCRP, and multidrug resistance-related proteins	

Several review articles have previously addressed nanoformulation approaches for tyrosine kinase inhibitors. None of these has exclusively focussed on orally administrated BCS class-II/IV second-generation TKIs, comprehensively compiling and comparing all nanoformulations that researchers have explored to address the solubility, permeability, P-gp efflux pump, and bioavailability challenge of these drugs; neither have proposed, after thorough critical analysis, potential formulations according to the criteria bioavailability enhancement, safety, long-term stability, scale-up readiness, clinical translational potential, and regulatory challenges. This review examines the current state and prospects of nanotechnology-enabled formulation strategies to overcome the biopharmaceutical challenges of second-generation TKIs. The scope of this review is a comprehensive study of various nanocarrier approaches to enhance the solubility and permeability of BTB, NTB, and DTB that have been explored recently, along with quality by design (QbD), regulatory challenges, translational status, and emerging trends in this class of drugs.

## Review

### Literature research methodology

**Figure 2:**
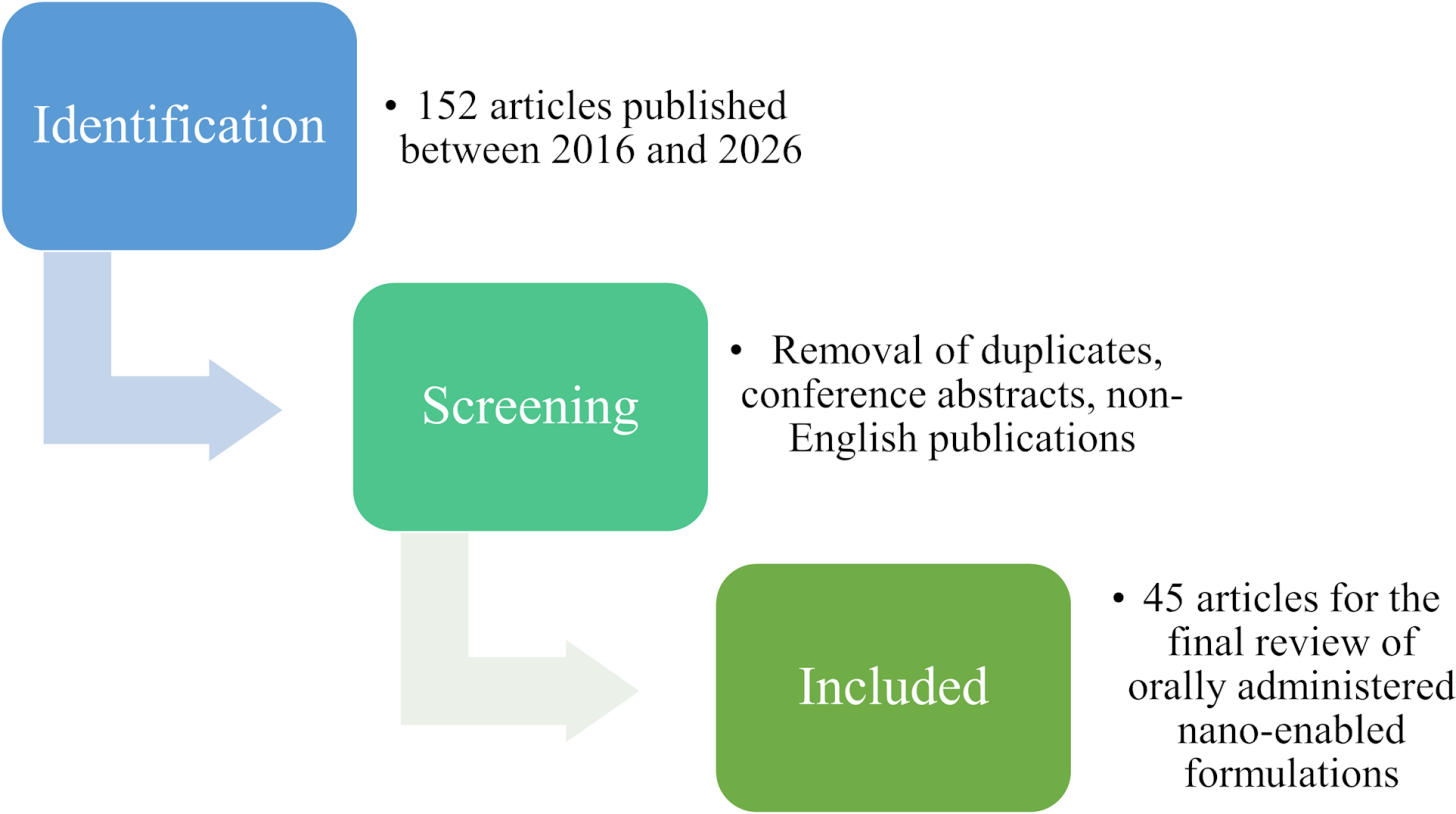
Systematic search methodology regarding nanotechnology-enabled second-generation TKIs.

The process of the literature search is depicted in Figure 2. The literature included in this review article was compiled through a systematic search of the scientific databases PubMed, Google Scholar, and Web of Science. Articles regarding nanotechnology-enabled formulations explored for the second-generation TKIs to increase their oral bioavailability, published between 2016 and 2026, were considered for inclusion. The search strategy used combinations of keywords including “dasatinib nanoformulation”, “bosutinib nanoformulation”, “nilotinib nanoformulation”, “oral bioavailability enhancement”, “TKI-nanoparticles”. Only articles published in English that reported formulation development, physicochemical and morphological characterization, solubility, permeability or pharmacokinetic or therapeutic performance evaluation of the nanotechnology-enabled delivery systems of TKIs were included in this review. Duplicate records, conference abstracts, non-English publications, parenteral route of administration, and studies lacking sufficient data were excluded from this review. The collected studies were critically analysed after summarization to list different nanoformulation strategies, their biopharmaceutical advantages, and their limitations. Out of the 152 articles collected during the initial search, only 45 were included after analysis based on the inclusion and exclusion criteria.

### Nanoformulations for solubility and permeability enhancement

**Figure 3:**
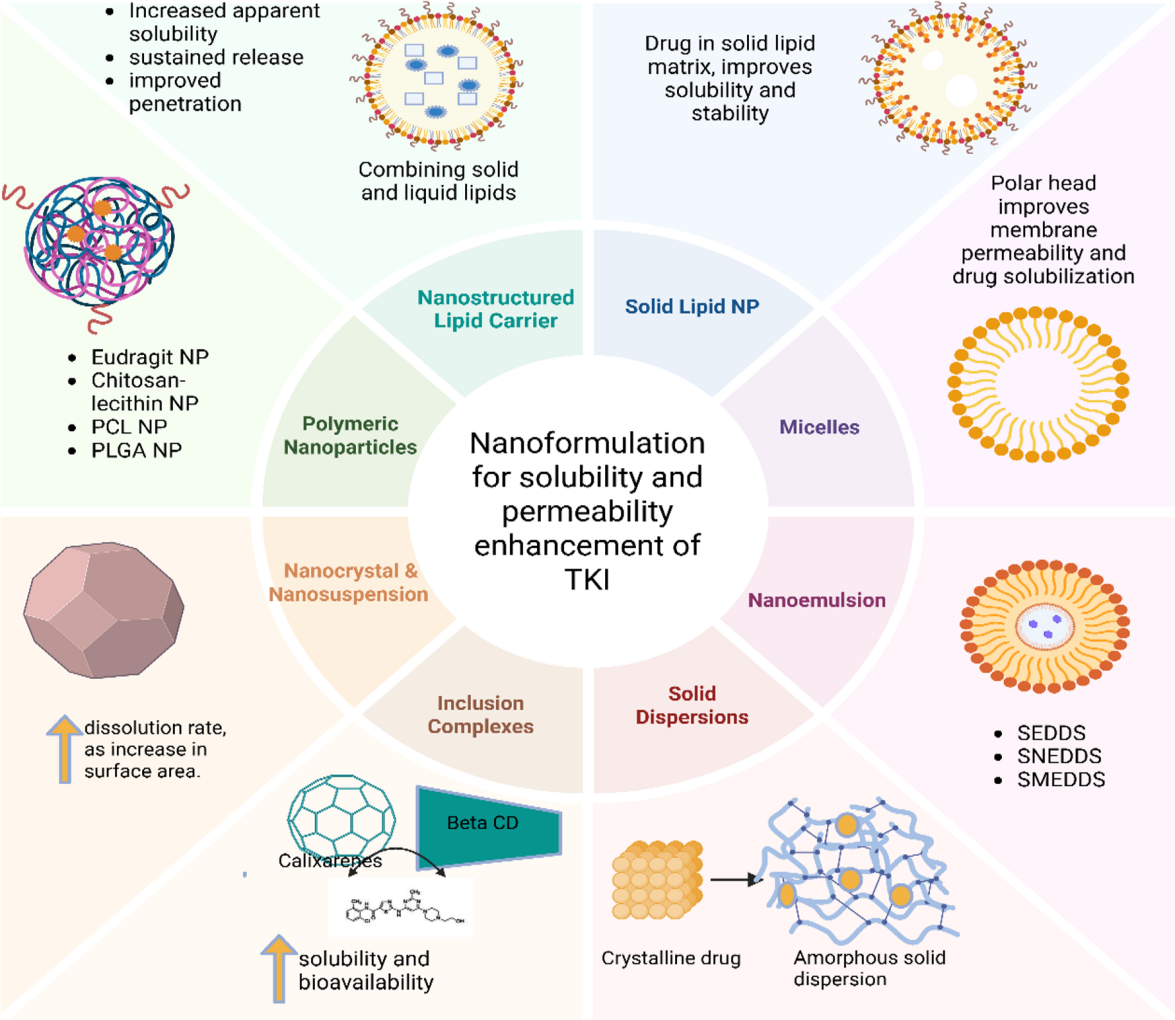
Nanotechnology Platforms for Enhancing Solubility and Permeability of TKIs. Figure 3 was created in BioRender. Mishra, B. (2026) https://BioRender.com/7ndce03. This content is not subject to CC BY 4.0.

Various researchers have explored a wide range of nanotechnological platforms to overcome the solubility and permeability limitations of second-generation TKIs, as summarized in Figure 3.

#### Solid lipid nanoparticles

Solid lipid nanoparticles (SLNs) are colloidal nanocarriers in which the drug is entrapped within a solid lipid matrix composed of biodegradable and biocompatible lipids. At the same time, hydrophilicity is imparted by an outer surfactant coating. Upon oral administration, they are mainly absorbed via lymphatic transport through chylomicron pathway and also improve the dissolution rate of the crystalline drug [19]. DTB-loaded SLNs were prepared using various lipids, including glyceryl tristearate, glyceryl tripalmitate, glyceryl trimyristate, soy lecithin, and poloxamer by the hot homogenization method. In the Caco-2 monolayers, *P*_app_ increased twofold and reduced P-gp efflux ratio due to the lipid-mediated membrane fluidization and inhibition of P-gp ATPase activity. In vivo evaluation of the optimised formulation demonstrated a 2.28-fold increase in bioavailability, attributed to amorphisation of the drug within the lipid matrix [20]. Another DTB-loaded SLN prepared with glycerol monostearate, poloxamer 407, and tyloxapol also demonstrated a 3.5-fold increase in the area under the curve (AUC) in the optimised SLN vs pure DTB, indicating improved oral bioavailability [21]. Similarly, NTB and BTB-loaded SLNs showed promising results with a particle size in the range of 187–198 nm, entrapment efficiencies of 85–86%, and a three- to fourfold increase in dissolution with sustained release for prolonged time [22–23]. Moreover, a 2.28-fold increase in peak plasma concentration and in mean residence time (MRT) of BTB SLN was observed when compared with the drug suspension [24]. SLNs formulated for the co-delivery of hesperidin and DTB demonstrated enhanced anticancer activity, reducing IC_50_ from 33.97 µg/mL (of DTB) to 4.03 µg/mL (of SLN) [25]. Consequently, this scalable SLN system, with a biocompatible solid lipid matrix made up of lipid excipients that are generally regarded as safe, provides advantages like lymphatic absorption and P-gp inhibition; also, it enables the conversion to solid-dosage form for oral administration by techniques like spray drying or freeze drying, and protecting the gastric mucosa from the side-effects of these TKIs [26]. However, associated gastrointestinal (GI) irritation due to the use of hydrophilic surfactants, lipid instability, poor drug loading capacity, and possible drug expulsion during storage due to polymorphic transition of the lipid are key challenges [27] that may hinder the long-term translational applicability compared to nanostructured lipid carriers.

#### Nanostructured lipid carriers

Formulation of nanostructured lipid carriers (NLCs) is another approach to address the poor aqueous solubility and limited bioavailability of second-generation TKIs, while overcoming the instability problem of SLNs by combining solid and liquid lipids. Liquid lipids can form an amorphous core that encapsulates drug molecules without the risk of expulsion, and studies have shown that second-generation TKI NLCs are stable for long-term use (over six months under accelerated conditions). NLCs composed of glycerin monostearate, Miglyol 812 N, and Solutol^®^ HS 15 loaded with DTB have shown a more than 1220-fold increase in saturation solubility, sustained release (25.14 ± 10.57% in 24 h), and enhanced tissue penetration, as observed by fluorescence assay, resulting in superior pharmacological activity compared with conventional formulations [28]. NTB-loaded NLCs made up of liquid lipid Caproyl^®^ PGMC, solid lipid Gelot^®^64, and emulsifying surfactant Sween 20 exhibited a high entrapment efficiency (EE) of 90.5%, a nanoscale size of 127.01 nm, a polydispersity index (PDI) of 0.258, and a 3.3-fold increase in peak plasma concentration (*C*_max_) compared with a free drug suspension, confirming the controlled and sustained release of the drug from NLCs [29]. Moreover, NLCs made of Tween 80/polaxamer 188 demonstrated P-gp inhibitory activity in Caco-2 studies, reducing the efflux ratio, which could be due to the competitive inhibition of P-gp ATPase by the excipients [30]. Another study investigating the potential of NLCs loaded with NTB reported positive results with optimised NLCs demonstrating a 1.86-fold increase in relative bioavailability compared with the commercial product and a 2.55-fold increase compared with the NTB suspension [31]. In parallel, lipid-based nanoformulations of BTB have demonstrated improved solubilisation and delivery of this highly lipophilic TKI, supporting the broader concept that lipid nanocarriers can mitigate dissolution-limited absorption and variability. BTB-loaded NLCs were formulated with a mean particle size of 131.7 nm and a PDI of 0.105. Pharmacokinetic evaluation of the optimised NLCs demonstrated a 2.6-fold increase in bioavailability under fasting conditions and a 1.6-fold increase under fed conditions [32]. NLCs, made by incorporating emulsifying liquid lipids, offer a high drug-loading capacity and superior stability compared to SLNs and can improve the bioavailability of poorly water-soluble drugs via lymphatic circulation. However, formulation complexity, cost-intensive processing equipment, the requirement for optimization studies, and reproducibility during large-scale manufacturing are associated challenges. A suitable and compatible surfactant or polymer is needed to impart a negative or positive charge on the surface of the NLCs, thereby preventing precipitation and making nanoformulations stable in the long term. Additionally, surfactant and polymer-related toxicity must also be considered for clinical translation [33].

#### Polymeric nanoparticles

Polymeric nanoparticles are versatile carriers to increase the solubility of poorly soluble drugs. The drugs are either encapsulated (nanospheres) or surface-adsorbed (nanocapsules) in the polymer matrix. NTB-loaded nanoparticles were formulated using Eudragit RL 100 and Eudragit RS 100 as polymers via the precipitation method. The resulting nanoparticles were stable and exhibited a mean particle size of 42.54 nm, a PDI of 0.218, and an EE of 99.50% [34]. Other natural polymer-based systems have also been explored, for instance, NTB-loaded bovine serum albumin (BSA) nanoparticles designed for bone marrow targeting demonstrated enhanced drug accumulation in bone tissue compared with the pure drug [35]. More recently, strategies aimed at prolonging intestinal residence time, such as self-assembled DTB-loaded lecithin–chitosan hybrid nanoparticles, showed a fivefold increase in intestinal absorption in in vivo studies relative to the pure drug. The enhanced performance was attributed to mucoadhesive interactions that prolonged residence time at the absorption site, while the nanoscale size facilitated improved intestinal permeability [36].

Poly(ε-caprolactone) (PCL), owing to its biodegradability, biocompatibility, low cost, and favourable drug permeability, has been widely explored for the delivery of TKIs. PCL-based systems can form endo-lysosomal vesicles, thereby enhancing intracellular drug delivery and anticancer activity. In this context, wool-like hollow PCL nanoparticles with a pH-sensitive NTB core and an enzyme-sensitive imatinib shell exhibited 64.65 ± 0.06% encapsulation efficiency and drug loading of 1.53 ± 0.02%, along with improved and controlled drug release [37]. To enhance the pharmacokinetic profile, protect the drug from degradation, and prolong its therapeutic action, DTB was encapsulated in poly(styrene-*co*-maleic acid) micelles. In vivo studies demonstrated an IC_50_ value of 0.09 for SMA-DTB vs 0.21 for free DTB, suggesting superior cellular uptake and anti-tumour activity against triple-negative breast cancer compared with the pure drug [38]. The integration of inorganic materials has also been explored to enhance the anticancer activity of TKIs. For example, magnetic core/polymeric shell nanoparticles have been developed in which graphene oxide–magnetic nanoparticle sediments were sequentially coated three times with a poly(lactic-*co*-glycolic acid) (PLGA)-based polymeric gel containing NTB, polyvinylpyrrolidone, and polyethene glycol (PEG). The resulting core–triple-shell nanoparticles exhibited sustained, gradual drug release over a prolonged period [39]. Poly(lactic-*co*-glycolic acid) is one of the most widely used Food and Drug Administration (FDA)-approved biocompatible polymers in polymeric nanoparticle-based drug delivery systems as its ester hydrolysis provides predictable biphasic drug release, governed by the polymer’s molecular weight and lactic acid/glycolic acid ratio; the uptake of such delivery systems occurs via clarithrin-mediated endocytosis, bypassing the P-gp efflux transporter. DTB-loaded PLGA nanoparticles were prepared using a double emulsion solvent evaporation method with Pluronic F68 and dimethyldioctadecylammonium bromide (DMAB) as stabilisers, resulting in average in vitro cumulative percentage releases of 88% and 73%, respectively, in 24 h [40]. Another biodegradable and biocompatible polyester, poly(cyclohexene phthalate), was used to synthesise DTB-loaded nanoparticles by nanoprecipitation to achieve controlled drug release and overcome its low aqueous solubility. The formulation exhibited good stability in 10% human plasma, while in vitro release studies demonstrated sustained drug release for up to 60 h. Furthermore, no cytotoxicity was observed in non-tumorigenic human embryonic kidney cells (HEK-293) and MCF10A cell lines, supporting the safety and potential applicability of this delivery system [41]. Hence, polymeric nanoparticles are versatile carriers that improve the therapeutic performance of TKIs by enabling targeted delivery, facilitating surface modification, prolonging intestinal residence time, providing sustained release, and inhibiting/bypassing efflux transporters. Moreover, their drug-loading capacity is higher than that of NLCs, and the presence of previously approved polymeric microparticles suggests strong potential for clinical translation of TKI-loaded polymeric nanoparticles. Nevertheless, issues such as residual organic solvents (e.g., acetone and dichloromethane), polymer-associated toxicity (depending on the concentration used), and high polymer costs continue to limit their widespread clinical adoption. In fact, batch-to-batch reproducibility and regulatory approval for complex polymeric systems remain challenging [42–43].

#### Nanocrystals and nanosuspensions

Nanocrystals and nanosuspensions, unlike other nanotechnology-enabled formulations, contain drug nanoparticles stabilized by surfactants rather than incorporating the drug into nanocarriers. Conceptually and mechanically, they are the simplest systems and have been shown to enhance the bioavailability of poorly water-soluble drugs by increasing their dissolution rate (exploiting relations described by the Ostwald–Freundlich and Noyes–Whitney equations) and minimizing food effects. Nanocrystals can overcome particle agglomeration issues commonly associated with other nanoformulations and may reduce first-pass metabolism by promoting lymphatic uptake [44]. In this context, DTB nanocrystals and nanoemulsions prepared using a high-gravity technique in rotating packed-bed reactors demonstrated, respectively, 1.63 and 1.35 times improved in vitro drug permeability compared to raw DTB and cytotoxicities of 30.08% and 22.33% vs 11.51% in M.D. Anderson metastatic breast cancer – 231 (MDA-MB-231) cells [45]. Likewise, DTB nanocrystals prepared by sonoprecipitation, using Tween 80 and Poloxamer 407, demonstrated a mean crystal size of 151 nm and a 2.2-fold higher saturation solubility [46]. Fucoidan-based, cubic BTB nanocrystals prepared by the nanoprecipitation technique as a dry powder for pulmonary delivery exhibited a tenfold increase in surface area and saturation solubility of BTB [47]. Amorphous nanosuspensions contribute to low lattice energy, increased apparent solubility, high membrane flux, and increased dissolution kinetics. A stabilized amorphous nanosuspension of the brick dust molecule (a class of molecules having strong crystalline lattice energy) NTB was prepared by an acid–base neutralization approach, where weakly basic NTB is solubilised in the acidic stabiliser solution, followed by neutralization in the alkaline medium, leading to precipitation of the drug in the amorphous form and avoiding recrystallization due to being enclosed in the micelles of the amphiphilic stabilizer. The XRD pattern confirmed the amorphous nature of NTB in the nanosuspension. There was a 36-fold enhancement of solubility and a 1.46-fold increase in the bioavailability of the NTB nanosuspension [48]. Overall, both nanocrystal and nanosuspension platforms increase the drug’s surface area, without requiring large quantities of carrier excipients (only trace amounts of FDA-approved stabilizers), leading to substantial improvements in TKI dissolution profiles without toxicology screening of excipients. This is one of the translational advantages over other nanotechnology-enabled platforms, as evidenced by the presence of marketed oral nanocrystal tablets (Rapamune^®^) and capsules (Emend^®^) after regulatory approval [49]. However, compared with other nanotechnology-enabled formulations, nanocrystals in aqueous media may undergo Ostwald ripening to give larger particles (>650 nm) [50]. This can compromise the formulation’s physical stability, limiting long-term therapeutic performance and reproducibility [51]. However, it can be avoided by the use of an effective surfactant or stabilizer or a combination of the two. Therefore, the nanocrystals should be properly evaluated for potential change in crystal form (polymorphism), Ostwald ripening, and rapid recrystallization during processing or after administration/dissolution, driven by the high interfacial energy of nanoscale drug particles [52].

#### Cyclodextrin complexes and inclusion complexes

Cyclodextrins (α-, β-, and γ-cyclodextrin, as well as surface-modified derivatives) are not conventional nanoformulations; but they can form nanoscale complexes and are hence discussed here as a nanotechnology-enabled solubilization carriers. Cyclodextrins are natural cyclic oligosaccharides composed of glucose units linked by α-1,4-glycosidic bonds, forming a hydrophobic inner cavity (inner diameter 6.0–6.5 Å) and a hydrophilic outer surface. These structures enable the formation of 1:1 or 1:2 host–guest inclusion complexes with hydrophobic TKIs via hydrophobic interactions, thereby enhancing their aqueous solubility and stability through interactions with the outer hydroxy groups. The aromatic rings of NTB, DTB, and BTB fit within the cyclodextrin cavity, and hydroxypropyl (HP) and sulfobutylether β-cyclodextrins preferably increase the solubility of the drugs for oral use compared to unmodified β-cyclodextrins [53]. A DTB–HP-β-cyclodextrin inclusion complex was prepared using a mechanochemical approach, in which the piperazine ring of DTB interacted with the cyclodextrin cavity, with the complexation efficiency exceeding 90%. This strategy enhanced bioavailability and solubility (15–40-fold), enabling controlled drug release and minimising first-pass metabolism, thereby reducing dosing frequency and lowering systemic exposure [54]. These cyclodextrin inclusion complexes can be further incorporated into liposomes, nanogels, micelles, nanofibres, nanocapsules, nanosponges, and nanoparticles, facilitating clinical translation and enabling potential therapeutic applications [55]. Host–guest inclusion complexes of NTB and DTB with calix[*n*]arenes, investigated using multilevel in silico docking, demonstrated enhanced bioavailability profiles for these TKIs. Calixarenes possess distinct upper and lower rims along with a hydrophobic cavity, enabling efficient complex formation while exhibiting low toxicity and good chemical stability [56]. Evidently, through host–guest interactions, these complexes increase the aqueous solubility and stability of the hydrophobic TKIs. Moreover, these cyclodextrins have FDA-approved oral applications, suggesting that the regulatory pathway is well-characterized and the complexation kinetics are scalable via simple co-precipitation. Despite its major advantages, there are challenges like dynamic equilibrium (endogenous substances like cholesterol can displace the drug from the host cavity, thereby reducing the solubilisation effect) [57], the need for optimised complexation constants (if it is too low, the complex would be unstable inside the body, if it is too high, the drug would not be released from the host molecule after absorption), and limited supersaturation maintenance (if precipitation inhibitors are not added). All these limitations have to be carefully addressed for successful clinical translation [58].

#### Solid dispersions

Although amorphous solid dispersions are not true nanosystems, unlike the other formulations discussed, they are included here as they can form nanoscale drug clusters upon dissolution, which is similar in mechanism of other nanoformulations indicated for bioavailability enhancement. Hence, they are used to molecularly disperse the poorly soluble active pharmaceutical ingredient (API) in the hydrophilic polymer or carrier matrix in its amorphous form. To prevent the amorphous form of a drug from reverting to its thermodynamically stable crystalline state, it can be converted into a solid dispersion (SD) by entrapping the drug within a polymer matrix with a higher Gibbs free energy than the crystalline form, thereby providing a greater driving force for dissolution. The amorphous drug is molecularly dispersed in the polymer using various formulation techniques [59]. In this context, NTB solid dispersions were prepared via spray drying using different polymers, namely Kollidon^®^, Soluplus^®^, and Lutrol^®^ F68. Dissolution studies conducted in simulated intestinal fluid (SIF), simulated gastric fluid (SGF), and pH-shift systems revealed that a 1:7 (NTB/Soluplus^®^) ratio was optimal and provided 630-fold greater solubility than crystalline nilotinib. Scanning electron microscopy (SEM) images further confirmed micelle formation within the solid dispersion system [60]. DTB solid dispersions were prepared using a zein–hydroxypropyl methylcellulose (HPMC) polymer system and subsequently compressed into tablet form. Zein, an adhesive matrix-forming polymer, enhanced the pH-dependent solubility of DTB (79.70% in vitro dissolution in fed-state simulated gastric fluid), thereby improving its dissolution behaviour [61]. In another study, cellulose acetate butyrate (CAB) was employed as a carrier to prepare an amorphous solid dispersion (ASD) of DTB. CAB helps in stabilizing the amorphous form of the drug. There was a 3.7–4.9-fold increase in dissolution, while time to peak drug concentration (*T*_max_), *C*_max_, and AUC were, respectively, 4.3-fold faster and 2.0- and 1.5-fold higher than those of the drug [62]. ASDs are highly effective at preventing drug recrystallization, thereby significantly improving bioavailability by molecularly dispersing the drug within the stabilizing polymers. Moreover, the FDA has published specific guidelines for the characterization of ASD pharmaceutical. Still, a few limitations persist: establishing drug–polymer miscibility before formulation; hygroscopic polymers have limited use as they can accelerate crystallization; no inhibition of P-gp; and system destabilization if high drug loading is used [59]. Solid dispersions can be loaded in nanofibres for localized drug release and nanogels for release at the target site, forming novel nanodrug delivery platforms with better drug transport and therapeutic potential.

#### Self-emulsifying drug delivery system

Self-emulsifying drug delivery systems (SEDDSs) are well-established platforms for enhancing the solubility and permeability of poorly soluble drugs by improving drug solubilisation, reducing particle aggregation, enhancing wettability, and promoting lymphatic absorption. These systems typically comprise an oil phase and one or more surfactants. Advanced subsets of SEDDSs (size > 300 nm), that is, self-microemulsifying drug delivery systems (SMEDDSs, <250 nm) and self-nanoemulsifying drug delivery systems (SNEDDSs, <100 nm) are also discussed in this section. Self-emulsification occurs due to a change in entropy [63]. Owing to their simple, scalable, and cost-effective industrial preparation, SEDDSs exhibit higher translational potential than many other novel drug delivery systems, particularly for BCS class-IV drugs [64]. DTB-loaded SEDDSs were formulated using cinnamon oil, selected for its high DTB solubilisation capacity, along with Tween^®^ 80, ethanol, and PEG 200. The dissolution efficiency increased from 56% for the pure drug to 93% for the SEDDS formulation. In situ intestinal absorption studies demonstrated that the fraction of drug absorbed per unit of intestinal length was 1.7–2.4 times higher with the SEDDS than with an aqueous drug solution. This could be due to P-gp inhibitory effect of the SEDDS along with membrane fluidization and lymphatic uptake. Furthermore, in vitro antitumor efficacy evaluated using the solid Ehrlich carcinoma model showed a 47% reduction in tumour weight, indicating superior therapeutic performance of the SEDDS compared with the pure drug (27% reduction) and the negative control [65]. SMEDDSs form thermodynamically stable microemulsions spontaneously upon dilution. A NTB-loaded SMEDDS formulated with Capryol 90, Transcutol HP, and Tween 80 showed a twofold increase in relative bioavailability and systemic exposure compared with NTB suspension. Pharmacokinetic evaluation following single-dose administration in Sprague Dawley rats showed a *T*_max_ of 5.33 h for the SMEDDS and 1.6 h for the suspension, indicating sustained release of NTB from the SMEDDS formulation [66]. Further evolved versions of SEDDSs, such as SNEDDSs, form a kinetically stable nanoemulsion upon dilution due to the stomach’s natural motility (external energy). Mageshwaran et al. developed a supersaturable self-nanoemulsifying drug delivery system (Su-SNEDDS) incorporating polyvinylpyrrolidone K30 as a precipitation inhibitor to suppress crystal growth and enhance formulation stability in the GI environment. This approach resulted in a 13.5-fold increase in the aqueous concentration of DTB compared with a conventional suspension. In vitro Caco-2 cell permeation studies demonstrated a 5.4-fold improved permeability coefficient. At the same time, in vivo pharmacokinetic evaluation in male SD rats showed a 2.7-fold increase in AUC relative to DTB suspension [67]. These self-emulsifying systems offer greater industrial translational potential than other nanotechnology-enabled formulations, which are less stable, require costly excipients, and involve complex production methods. They spontaneously form fine emulsions (nanodroplets) that boost intestinal permeability and maintain higher drug concentration in the GIT by eliminating the dissolution rate-limiting step (as pre-dissolved) and inhibiting P-gp ATPase activity [68]. However, since the surfactant load is high, it can cause GI irritation, the drug can precipitate if supersaturation is not maintained upon GI dilution, and there is a possibility of high inter-individual variability due to dependence on GI lipase activity [69].

#### Micelles

Micelles are formed when surfactant concentrations exceed the critical micellar concentration (CMC); micelles comprise hydrophilic head groups oriented toward the aqueous phase and hydrophobic hydrocarbon tails forming the core. This architecture offers several biopharmaceutical advantages by enhancing drug solubilisation, modulating membrane permeability, and inhibiting P-gp via membrane fluidisation, thereby improving absorption. Surfactants spontaneously self-assemble into micelles above their critical micellar concentration, which can be further processed into conventional solid dosage forms, such as tablets, using appropriate excipients. In this context, DTB-loaded micelles were formulated using the non-toxic surfactant sodium lauryl sulfate (SLS) and subsequently spray-dried with lactose monohydrate to obtain microparticles. These spray-dried microparticles were granulated via a wet granulation process and compressed into tablets. In vitro dissolution studies demonstrated enhanced drug solubility from the micellar system. Furthermore, in vivo bioavailability studies in male Wistar rats confirmed increased systemic exposure, with AUC rising from 1381 to 1919 ng/mL/h and *C*_max_ increasing from 200 to 318 ng/mL [70]. Polymeric micelles are composed of amphiphilic polymers that form a hydrophobic core and a hydrophilic shell, enabling efficient encapsulation of lipophilic drugs. DTB-loaded micelles were prepared using a TPGS–Soluplus copolymer via the thin-film hydration technique. TPGS, a vitamin E derivative, is an FDA-approved excipient known to inhibit the P-glycoprotein efflux transporter. In vitro cytotoxicity studies conducted in the HepG2 cell line demonstrated enhanced therapeutic efficacy, supporting improved oral bioavailability. Furthermore, in vivo pharmacokinetic evaluation in male Wistar rats, using DTB solution and blank micelles as controls, revealed a 2.16-fold increase in AUC and a 1.3-fold prolongation of MRT [71]. These systems have the potential to enhance drug solubility and absorption while improving therapeutic efficacy. However, these micelles come with their own challenges. Thermodynamic instability occurs when surfactants exist only in micellar form above their CMC [72]. A single excipient like Pluronic/Cremophor/TPGS is used, which requires a high concentration, leading to the risk of GIT toxicity. In fact, this liquid formulation cannot be easily converted to a solid dosage form, which poses storage and stability issues [73].

### Multifunctional and targeted nanosystems

Multifunctional and targeted nanosystems incorporating second-generation TKIs have been extensively explored for the treatment of various cancers to enhance therapeutic efficacy, enable site-specific delivery, and minimize adverse effects [74]. Soghrati et al. developed co-loaded DTB and miR-30a liposomes targeting the neuropilin-1 receptor for the treatment of triple-negative breast cancer. The CRGDK peptide was employed as a targeting ligand. Lipopolyplexes were first formed by condensing miR-30a with a cationic copolymer, followed by encapsulation into liposomes using the thin-film hydration technique. The resulting targeted liposomes demonstrated superior inhibition of cell proliferation and migration, along with enhanced cellular uptake, when evaluated in MDA-MB-231 cells [75]. Hyaluronic acid-conjugated and curcumin TPGS were employed to formulate pH-sensitive micelles for the delivery of DTB to tumour cells. These nanoparticles exhibited enhanced drug release under acidic conditions characteristic of the tumour microenvironment and demonstrated good in vitro stability. In vivo near-infrared (NIR) fluorescence imaging confirmed effective tumour targeting. At the same time, additional in vivo studies in BALB/c nude mice showed reduced systemic toxicity associated with the free drug and significant inhibition of tumour growth [76]. Similarly, ligands such as hyaluronic acid can be employed to target the CD44 receptor, which is overexpressed on tumour cells. Accordingly, hyaluronic acid-decorated, self-assembled DTB-loaded nanoparticles exhibited high targeting specificity and enhanced cytotoxicity in human nasopharyngeal epithelial carcinoma cell line (HNE1) cells [77]. Biotin-modified BTB-loaded liposomes were developed to target estrogen receptor-positive cancers, exploiting biotin receptor-mediated uptake via the sodium-dependent multivitamin transporter. The IC_50_ value of biotin–BTB liposomes was 3.2 µM, the lowest among the tested formulations, indicating enhanced anticancer activity at reduced drug doses. This finding confirmed that biotin functionalization promoted greater intracellular retention of BTB in MCF-7 cells. Biodistribution studies further demonstrated prolonged accumulation of the liposomes at the tumour site for up to 24 h. Moreover, pharmacokinetic parameters, including AUC, MRT, and *C*_max_, were significantly higher for biotin-BTB liposomes than for pure BTB and non-targeted BTB liposomes, indicating enhanced systemic exposure. But liposomes face major clinical challenges, namely physical and chemical instability, difficulty in scale-up, and a low drug-to-lipid ratio [78].

DTB-loaded multifunctional micellar nanoparticles prepared using polyethene glycol (5000 Da)–phosphoethanolamine and folic acid–polyethylene glycol (2000 Da)–phosphoethanolamine were designed to target matrix metalloproteinase-2 (MMP-2) and folate receptors, thereby improving tumour specificity and anticancer efficacy. Evaluation in three-dimensional multidrug-resistant (MDR) tumour spheroids demonstrated enhanced tissue penetration of DTB. Furthermore, in a melanoma xenograft mouse model, dual-targeted micelles exhibited prolonged systemic circulation, reduced accumulation in non-tumour tissues, and increased tumour localisation compared with non-targeted formulations [79]. Similarly, active targeting of hepatic cancer cells by folate-functionalized BTB-loaded cubosomes reduced off-target side effects by enhancing cubosome uptake via folate receptor-mediated endocytosis [78]. Cancer-derived hybrid exosomes, which were PEGylated after exosome isolation and loaded with DTB, demonstrated enhanced cellular uptake due to the presence of exosomal surface markers such as CD9 and CD81. Additionally, pH-sensitive drug release in the acidic tumour microenvironment promoted preferential release at the tumour site. Collectively, these features enhanced the cytotoxic activity of DTB against pancreatic cancer cells [80]. As a potential multifunctional delivery strategy, BTB-loaded high-density lipoprotein (HDL) nanoparticles were formulated using the film hydration technique to exploit their radiosensitising potential in tumours. This biomimetic system enables natural tumour targeting via the scavenger receptor class B type 1 (SR-B1), which is overexpressed on tumour cells and tumour-associated macrophages. An apolipoprotein A-1 mimetic peptide solution was used as the hydration medium. In vitro cellular uptake and radiosensitisation studies conducted in squamous cell carcinoma cell line (UM-SCC-1) cells demonstrated effective cell cycle disruption, reduced cell proliferation, and decreased intracellular ATP levels. Furthermore, in vivo evaluation in a mouse oral squamous cell carcinoma (MOC-1) tumour-bearing syngeneic murine model using C57BL/6 mice demonstrated the highest tumour growth inhibition, confirming the formulation’s enhanced radiosensitising efficacy [81]. All these suggested the advantage of surface functionalized nanoplatforms for improving the delivery of TKIs (Table 3).

**Table 3:** Nanosystems developed for second-generation tyrosine kinase inhibitors.

Drug	Nanosystem type	Physical attributes^a^	Primary performance gains	Ref.

bosutinib	HDL nanoparticles	size: 8.9 ± 0.6 nm; DL: >10%	radiosensitisation; enhanced tumour growth inhibition	[81]
bosutinib	lipid NPs (quality by design optimised)	size: 124.95 nm; EE: 89.387%; DL: 4.56 ± 0.23%	2.6-fold oral bioavailability increase in fasted state and 1.36 fold increase in the fed-state; reduced food effect	[32]
bosutinib	folate conjugated cubosomes	size: 188.5 ± 2.25 nm; EE: 90.31 ± 3.15%; DL: 14.9 ± 2.03%	targeted hepatic delivery; controlled release; improved pharmacokinetics (PKT)	[82]
bosutinib	fucoidan-based nanocrystals	size: 150 nm	tenfold increase in surface area and saturation solubility	[47]
bosutinib	biotin-conjugated liposomes	size: 257.73 nm; EE: 87.78%	38-fold AUC increase; improved anticancer activity/safety	[78]
bosutinib	SLNs	size: 150.73 ± 4.47 nm; EE: 93.56 ± 0.30%; DL: 4.8%	enhanced lymphatic transport; improved bioavailability	[83]
nilotinib	SLNs	size: 187–198 nm; EE: 85–86%, D/L ratio 0.16:1	fourfold solubility enhancement; central composite design optimised release	[23]
nilotinib	magnetic core–shell NPs	size: 70–100 nm; EE: 80–90%	60 h-controlled release; sustained drug delivery	[39]
nilotinib	amorphous nanosuspension	size: 130.5 ± 1.22 nm; EE: N/A; 60 mg NTB in 0.3% soluplus and 0.4% HPMC	36-fold enhanced solubility; 1.46-fold higher relative BA	[48]
nilotinib	polymeric NPs	size: 42.54 nm; EE: 99.50%; DL: 22.3%	enhanced solubility and dissolution rate	[34]
nilotinib	BSA NPs	size: 111 nm; EE: 85–90%; DL: 8.07 ± 0.65%	prolonged release; high biocompatibility	[84]
nilotinib	solid dispersions	size: 100–500 nm; EE: N/A; D/P ratio 1:7	630-fold enhancement in solubility in simulated intestinal fluid	[60]
nilotinib	SMEDDS	size: 8.5–10.56 nm; EE: N/A; DL: 0.5%	twofold increase in systemic exposure; 87% release	[66]
nilotinib	wool-like PCL NPs	size: 271.3 nm; EE: 55.26%	synchronised dual drug delivery; synergistic effect	[37]
dasatinib	nanoemulsion	size: 84 ± 10 nm; EE: 83%	30-fold tumour selectivity; 60% drug release	[65]
dasatinib	MMP-2/folate micelles	size: 100–150 nm; EE: 75–85%; DL: 2.47%	fourfold uptake in MDR cells; deep spheroid penetration	[79]
dasatinib	lecithin–chitosan hybrid	size: 150–250 nm; EE: 80–88%	mucoadhesion; 5.08-fold increase in AUC	[36]
dasatinib	β-cyclodextrin complex	size: 5–10 nm; EE: 85–95%; DL: 10%	controlled release; enhanced stability	[36]
dasatinib	hyaluronic acid-TPGS micelles^b^	size: 66.14 ± 4.02 nm; EE: 67.09%; DL: 5.94 ± 0.23%	pH-responsivity; CD44-targeted release	[76]
dasatinib	SLNs	size: 162.3 nm; EE: 93%	IC_50_ reduced from 33.97 to 4.03 µg/mL; release prolonged to 48 h	[25]
dasatinib	nanocrystals	size: 200–750 nm	fourfold higher solubility; increased cytotoxicity	[45]
dasatinib	nanoemulsion	size: 16.15 ± 0.45 nm	enhanced dissolution rate and membrane flux	[45]
dasatinib	polymeric NPs	size: 82.23 ± 1.07 nm; DL: 2.1%	accumulation of the drug reverses MDR and inhibits P-gp	[77]
dasatinib	PLGA nanocarriers	size: 123 nm; EE: 80%	sustained release; enhanced stability	[40]
dasatinib	lipopolyplexes	size: 97.20 ± 0.99 nm; EE: 99.65%	superior cellular uptake; targeted action	[75]
dasatinib	SLNs	size: 150 nm	89% cytotoxicity; 2.28-fold improved BA	[20]
dasatinib	polymeric micelles	size: 74.477 nm; EE: 64.479%; DL: 5.05 ± 1.01%	MRT 1.3-fold longer; AUC 2.6-fold higher	[71]
dasatinib	SMA micelles	size: 198 nm; EE: 65%; DL: 11.5%	protection from degradation; tumour internalisation	[38]
dasatinib	ASD tablets	–	45% BA; 30% reduction in dose vs crystalline	[85]
dasatinib	Su-SNEDDS	–	13.5-fold higher aq concentration; 2.7-fold AUC	[65]
dasatinib	zein-HPMC SD tablets	size: 107.3–190.5 nm; EE: N/A; D/P 2:3	79–90% drug release in biorelevant media	[61]
dasatinib	SEDDS (microscale)	size: 2040–7119 nm; EE: 91–97%	enhanced cytotoxic effect in cancer lines	[86]
dasatinib	micelles in microparticles	size: 120–140 nm; EE: N/A	water solubility; faster release; increased *C*_max_/AUC	[70]
dasatinib	poly(cyclohexene) phthalate NPs	size: 113–100 nm; EE: 96.7 ± 0.8; DL: 10.1 ± 0.4%	quicker release; superior efficacy	[41]
dasatinib	hybrid exosomes	size: 119.90 nm; EE: 88.7%	enhanced cellular uptake; CD9/CD81 exosomal markers	[80]

^a^Drug loading (DL) is expressed relative to the total excipient mass [(API mass/total excipient mass)×100].

### Comparative analysis of nanotechnology-enabled formulations for second-generation TKIs

Table 4 compares nanotechnology-enabled formulations explored by various researchers for second-generation TKIs. Based on the performance metrics of all the analysed studies, nanotechnology-enabled formulations exhibit distinct biopharmaceutical advantages, but definitive comparison among them is limited due to the heterogeneity of the reviewed articles (as experiments have not been performed in identical environment across all the nanosystems) and pending clinical validation. After careful, comparative critical analysis, the studies suggest that, in the context of bioavailability enhancement, surface-modified liposomes demonstrated the highest increase in the individual studies, owing to the dual benefits of active receptor targeting and lipid-mediated absorption. But their complexity in formulation makes them unsuitable for scale-up for industrial production, and clinical translation and its superiority over other nanosystems cannot be established due to lower number of in vivo studies for comparison. In improving the solubility of brick dust molecules, amorphous nanosuspensions and nanocrystals are the most effective, as they lower the lattice energy. In the context of permeability enhancement, Su-SNEDDSs provide the highest measured permeability gain, but require the use of precipitation inhibitors to maintain stability. In the context of intestinal retention, evidently, mucoadhesive hybrid nanoparticles prolong the drug’s retention at the absorption site. But overall, based on the comprehensive multicriteria analysis of the studied nanoformulations and nanotechnology-enabled formulations for DTB, BTB, and NTB individually, ASDs stand out (when interpreted separately). The second best, in case of DTB, was found to be mucoadhesive chitosan-lecithin nanoparticles; for NTB nanocrystals and SMEDDSs, it was both; regarding BTB, it was biotin-conjugated liposomes and folate-conjugated cubosomes.

**Table 4:** Comprehensive comparison of nanotechnology-enabled delivery systems.

S.NO	Nanosystem category	Key advantages	Major challenges

1	amorphous solid dispersions (ASDs)	highest clinical success (FDA-approved); prevents recrystallisation; improved solubility and dissolution, industrial scalability	risk of converting back to the crystalline state over time, and moisture sensitivity
2	targeted lipid systems (liposomes/cubosomes)	exploits receptor-mediated uptake (biotin/folate); superior site specificity, reduces systemic toxicity	high manufacturing costs, complex regulatory hurdles, physical and chemical instability, and leakage during storage
3	SNEDDSs	enhances wettability and membrane flux; high translational potential	requires precipitation inhibitors as risk of precipitation; high surfactant concentrations, GI irritation
4	mucoadhesive hybrid NPs	prolongs residence time in the gut via mucoadhesion; facilitates absorption	complex multistep manufacturing; stability in gastric fluid
5	solid lipid nanoparticles (SLNs)	promotes lymphatic transport (bypasses first-pass metabolism), biocompatible, sustained release, high translational potential	limited drug loading; risk of drug expulsion during storage, polymorphic instability
6	nanostructured lipid carriers (NLCs)	high drug loading minimises fasted/fed variability**,** enhances tissue penetration, and reduces crystallinity	technical complexity in lipid-matrix optimisation; surfactant-associated toxicity
7	polymeric micelles	inhibits P-gp efflux (TPGS); deep tissue/spheroid penetration, controlled release, enhanced intracellular accumulation	risk of dissociation in circulation; generally lower drug loading, dilution-induced instability
8	nanocrystals/nanosuspensions	massively increases surface area; minimises food effects, high drug loading, simpler composition and enhanced dissolution rate	risk of particle agglomeration if not properly stabilised, physical instability
9	polymeric nanoparticles	controlled release, MDR reversal capability and enhanced intracellular accumulation	polymer toxicity, residual solvents, scale-up challenges
10	biomimetic/hybrid systems	exploits natural biological pathways (SR-B1 receptor); pH-sensitive release, tumour-targeting	extremely difficult to scale up for industrial production and complex characterisation
11	inclusion complexes	simple host–guest interaction; improves stability and dosing frequency	limited to drugs that fit the hydrophobic cavity

### In vitro and in vivo evaluation

#### In vitro solubility and in vitro release testing

Second-generation TKIs exhibit pH-dependent solubility and variability [87–88]. Therefore, an equilibrium solubility study of the nanosystem must be conducted across all biorelevant pH ranges. Solubility limits of the nanoformulations can be established and compared with those of the pure drugs and their conventional formulations using the dialysis bag method. In vitro release studies were conducted in different biorelevant media and formulations (pH 7.4, 0.1 M HCl, fasted-state simulated gastric fluid (FaSSGF)/fasted-state simulated intestinal fluid (FaSSIF), SGF/SIF) for all three drugs. Due to this variability in media, a direct comparison of different nanoformulations of a particular drug cannot be done.

**Bosutinib.** Panigrahi et al. used a pH 7.4 buffer containing 0.5% SLS as the dissolution medium and reported that only 20% of the pure drug was released over 60 h. In contrast, the drug-loaded lipid nanoparticles (LNPs) exhibited approximately 95% drug release within the same duration, with an initial burst release attributed to the unentrapped drug [22]. Similarly, BTB-loaded SLNs prepared using dynasan-118 as the lipid matrix exhibited a cumulative drug release of 80.55 ± 1.10% over 24 h under dissolution conditions of 0.1 N HCl for the initial 2 h followed by phosphate-buffered saline (PBS, pH 6.8) for the subsequent 22 h [83]. Even functionalized BTB-loaded liposomes demonstrated promising release behaviour, with 85.56 ± 0.95% of the drug released over 48 h in a pH 7.4 buffer containing 0.1% Tween [78]. Kumar et al. demonstrated that the saturation solubility of fucoidan-based BTB-loaded nanocrystals in simulated lung fluid was approximately tenfold higher than that of the coarse drug. Moreover, the in vitro dissolution rate increased markedly from 6.5% within 6 h for the pure drug to about 60% over the same duration for the nanocrystal formulation, with complete drug dissolution achieved within 48 h. These findings confirm the potential of fucoidan nanocrystals to enhance the solubility of this BCS class-IV drug significantly [47]. Additionally, LNCs of BTB effectively reduced variability in drug dissolution across gastric and intestinal pH conditions and under fed and fasted states [32].

**Nilotinib.** NTB-loaded BSA nanoparticles exhibited pH-dependent drug release, with cumulative release values of 84.5%, 59.7%, and 43.8% at pH 5.0, 6.0, and 7.4, respectively, after 24 h [35]; a similar study showed 98% release at 4.5 pH and complete release at pH 7.4 in 72 h [58]. The NTB-loaded SLNs exhibited approximately 90% drug release in SIF, which was markedly higher compared with the pure drug, showing only about 10% release under the same conditions [23]. The NTB brick dust nanosuspension exhibited a solubility of 259 µg/mL in FaSSIF and 359 µg/mL in FeSSIF. In contrast, the pure drug showed a markedly lower solubility of only 7.27 µg/mL. In addition, the dissolution rate of the nanosuspension was substantially higher than that of the pure drug, achieving 29.48% and 54.04% drug release in FaSSIF and FeSSIF, respectively, after 120 min [48]. Polymeric nanoparticles formulated with Eudragit showed a high drug release rate of 94–99% within 24 h in acetate buffer [24]. Even core–shell magnetic nanoparticles loaded with NTB exhibited a high cumulative drug release, ranging from 90% to 96.5% across various formulation combinations [39]. A SMEDDS of NTB showed 87% release in 24 h [66]. Collectively, the in vitro evaluation results highlight the effectiveness of nanotechnology-enabled delivery systems in enhancing the dissolution and solubility profiles of NTB across different media and pH conditions.

**Dasatinib.** DTB-loaded lipopolyplexes exhibited 88 ± 4.1% drug release in a pH 7.4 buffer containing 2% Tween 80 [74]. In addition, cocrystallisation of DTB with hydroxybenzoic acid resulted in a sixfold increase in aqueous solubility, as determined by the shake-flask method [89]. The micellar system demonstrated enhanced DTB release (82%) at pH 5.0 compared with pH 6.0 and 7.4, confirming the drug’s pH-dependent solubility [76]. Furthermore, Sokač et al. reported solubility enhancement via cyclodextrin inclusion complexation, with 79% drug release within 45 min [54]. DTB-loaded SLNs showed a rapid drug release of 66.5% within 24 h [25]. Similarly, lecithin–chitosan nanoparticles exhibited higher drug release in acidic media (79%) in SGF compared with SIF (53%), while both formulations performed better than the pure drug [26]. DTB nanocrystals and nanoemulsions exhibited markedly improved dissolution rates of 48.38% and 56.96%, respectively, whereas the pure drug showed only 4% dissolution in a pH 6.8 buffer after 24 h [45]. Furthermore, pH-responsive drug release from TPGS–hyaluronic acid–DTB nanoparticles was confirmed, with 56.75% release under acidic tumour-mimicking conditions compared with 44.93% at pH 7.4 [77]. DTB-loaded PLGA nanoparticles stabilised with DMAB released 88% of the drug within 24 h [40]. In contrast, TPGS–Soluplus micelles demonstrated sustained release, reaching approximately 80% only after 72 h. The solid dispersion tablets of DTB showed improved release, with more than 75% in the biorelevant medium, when compared to pure DTB tablets, which showed less than 15% release [61]. Similarly, SEDDSs achieved 83% drug release compared with 23% for the pure drug [60]. DTB entrapped in micellar nanoparticles exhibited rapid and nearly complete drug release (≈100%) within 45 min, whereas the pure drug showed only ≈20% release over the same period [70]. A similar enhancement in dissolution behaviour was observed with nanoemulsion formulations of DTB [64]. In addition, poly(cyclohexene phthalate)-based nanoparticles enabled controlled drug release, achieving up to 99% release over 60 h [41].

#### Cellular uptake and transport studies

A comprehensive evaluation of the cellular uptake and transport mechanisms of nanosystems developed to enhance the solubility and permeability of these TKIs is essential. Multiple uptake pathways have been proposed for these nanosystems, including phagocytosis, macropinocytosis, clathrin-dependent endocytosis, caveolin-dependent endocytosis, micellar transport, and passive diffusion. These mechanisms are largely influenced by the nanosystems’ size, morphology, and surface chemistry, which govern their interactions at the cellular level [90]. Accordingly, various in vitro models, including Caco-2 and HT29-MTX cell lines and lacteal cell models incorporating lymphatic endothelial cells, have been employed by researchers to investigate the transport and uptake of nanosystems [91]. To begin with, quantitative and qualitative cellular uptake studies in MDA-MB-231 cells using DTB-loaded lecithin–chitosan hybrid nanoparticles demonstrated enhanced nanocarrier permeation and increased intracellular drug concentration [36]. Similarly, permeability studies conducted on Caco-2 cell monolayers demonstrated that DTB nanoemulsions and nanocrystals exhibited significantly higher apparent permeability coefficients, *P*_app_, compared with the raw drug, with increases of approximately 1.63- and 1.35-fold, respectively [32]; SNEDDSs and Su-SNEDDSs of DTB exhibited 2.25- and 5.5-fold increases, respectively, in the apparent permeability coefficient across Caco-2 cell monolayers, along with enhanced cellular uptake of coumarin-6-loaded formulations [67]. DTB-loaded polymeric micelles showed enhanced accumulation in HepG2 cells within 1–4 h compared with the pure DTB [66]. Furthermore, methyl thiazolyl tetrazolium assays of DTB-loaded spray-dried micelles in the leukaemia Baf3/wt cell line showed an IC_50_ value of 287 ± 8 nM, which was lower than that of the pure drug, indicating enhanced inhibitory activity and improved cellular permeability [70]. Cellular uptake studies of NTB-loaded BSA nanoparticles in MCF-7 cells demonstrated rapid permeation within 2 h post-incubation and sustained intracellular retention for up to 24 h. Similarly, wool-like PCL nanoparticles of NTB, when incubated with leukaemia KU812 cells and healthy C13895 cells, showed time-dependent cellular uptake via clathrin-dependent endocytosis [37]. In addition, fluorescein isothiocyanate-labelled BTB-loaded liposomes exhibited high fluorescence intensity upon incubation with MCF-7 cells, confirming efficient cellular internalisation [78]. BTB-loaded LNCs exhibited enhanced cytotoxic activity in MCF-7 cells, indicating increased cellular permeability mediated by endocytic uptake, and demonstrated similar activity in Caco-2 cell lines. Notably, blank LNCs showed no significant cytotoxicity, confirming the safety of the carrier system [32]. Likewise, BTB-loaded SLNs (when evaluated in ATCC cell lines) achieved 89% cell inhibition compared with only 42% for BTB suspension [24]. In addition, BTB-loaded lipid nanoparticles (LNPs) displayed an IC_50_ value that was eightfold lower than that of the pure drug, along with time-dependent enhancement in cellular internalisation observed in MCF-7 cells [22].

#### In vivo pharmacokinetic studies

**Figure 4:**
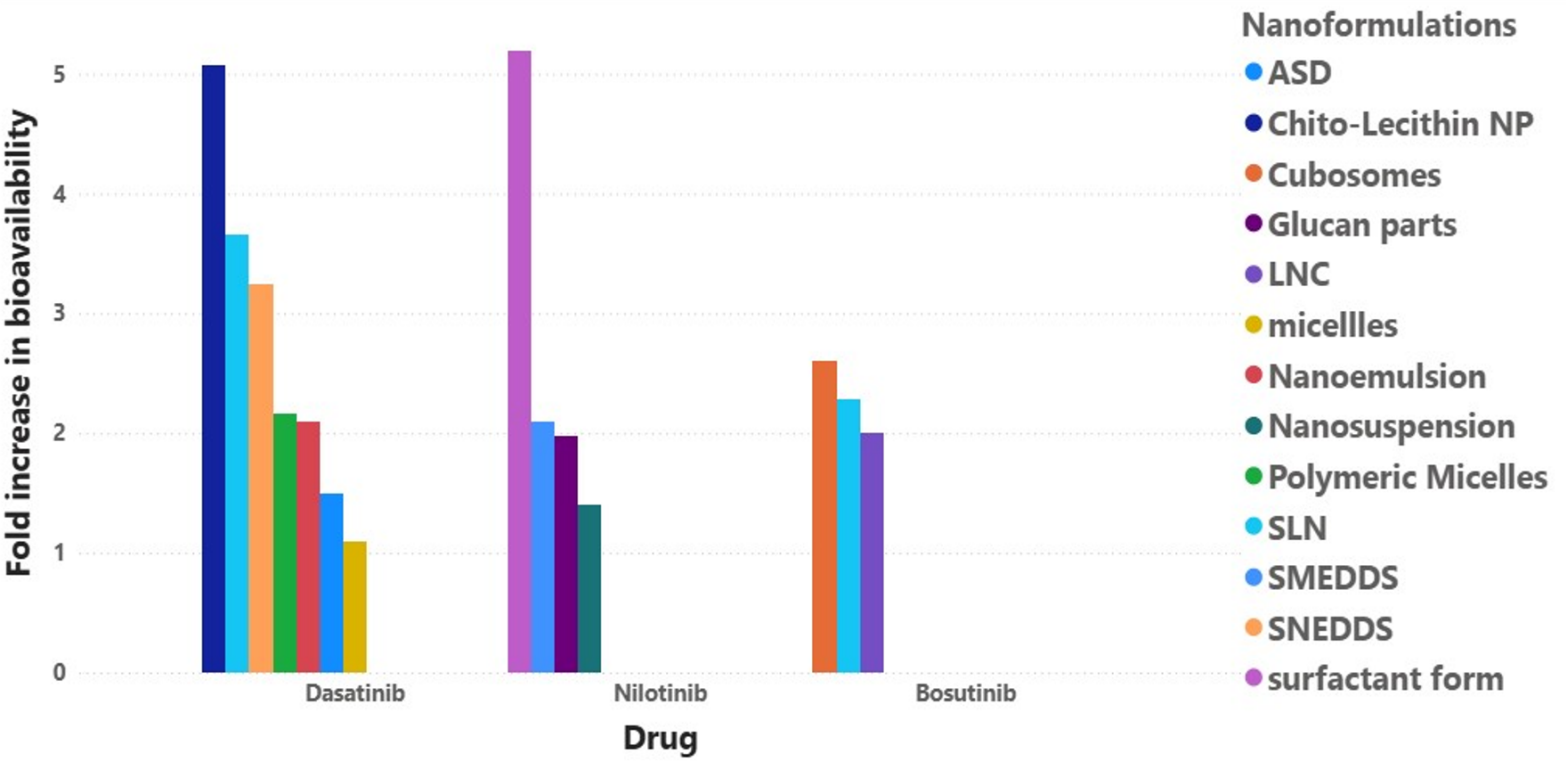
Bioavailability increase of nanoformulations when compared to the free drug.

In vivo animal models are widely employed to substantiate the improved pharmacokinetic performance of nanocarriers developed for second-generation tyrosine kinase inhibitors. The extent of bioavailability enhancement achieved by various nanosystems compared with the free drug is summarized in Figure 4.

**Bosutinib.** The pharmacokinetic parameters of BTB-loaded SLNs were evaluated in male Wistar rats following oral administration, demonstrating a 2.28-fold increase in bioavailability. This improvement was attributed to enhanced lymphatic transport, which helped circumvent first-pass metabolism and mitigate the drug’s low oral bioavailability [24]. Similarly, folate-functionalized BTB-loaded cubosomes exhibited prolonged mean residence time (MRT, 15.35 h), along with increased area under the curve (AUC, 1540.55 µg·h/mL) and peak plasma concentration (*C*_max_) in rat plasma, resulting in enhanced bioavailability [79]. The lipid nanocarriers of BTB also showed similar results, with 1.36- and 2.6-fold increases in bioavailability in the fasted and fed states, respectively, in SD rats [32]. Biotin-functionalized BTB-loaded liposomes exhibited the most pronounced improvement in pharmacokinetic performance among the evaluated nanosystems, with a *C*_max_ of 79.07 ± 5 µg/mL compared with 4.7 ± 1 µg/mL for the pure drug. Moreover, the AUC increased approximately 38-fold relative to the free drug, indicating a substantial enhancement in systemic exposure and plasma drug concentration over time [78].

**Nilotinib.** Single oral administration of brick dust amorphous NTB nanosuspensions in male SD rats demonstrated an improved plasma concentration–time profile, with a 1.5-fold increase in *C*_max_ and a 1.46-fold increase in AUC compared with crystalline NTB [48]. Comparable enhancements were observed with NTB-loaded SMEDDSs, which exhibited approximately a twofold increase in both AUC and relative bioavailability [66]. Yeast glucan particles were developed as carriers for NTB to enhance its bioavailability and were evaluated in rats using a crossover design with NTB capsules as the reference. Both *C*_max_ and AUC were approximately doubled, while lymphatic transport studies revealed a fourfold increase in *C*_max_ and a 2.7-fold increase in absolute lymph bioavailability, indicating that lymphatic uptake combined with drug amorphisation contributed significantly to bioavailability enhancement [66]. Additionally, in vivo evaluation of Tween 20-solubilised NTB in male rats showed a 5.2-fold increase in bioavailability compared with NTB suspension, attributed to enhanced solubility and digestibility [92].

**Dasatinib.** Oral administration of DTB-loaded SLNs to male Wistar rats resulted in a 2.28-fold increase in bioavailability compared with the marketed formulation [20]. A pharmacokinetic study of an ASD conducted in Albino New Zealand White rabbits (two males and two females) demonstrated faster drug absorption and more gradual elimination compared with the crystalline drug. The relative bioavailability of the ASD was 198.7%, representing a 145% increase over the pure drug. Furthermore, the pharmacokinetic parameters *T*_max_, *C*_max_, and AUC showed 4.3-, 2.0-, and 1.5-fold increases, respectively [62]. These findings are further corroborated by a clinical pharmacokinetic study conducted by Lennernäs et al. in healthy male subjects, in which oral administration of a DTB ASD was compared with the marketed tablet. The ASD formulation demonstrated a 2.3–4.8-fold increase in drug absorption, reduced inter- and intra-subject variability in pharmacokinetic parameters, and achieved bioequivalence at a 30% lower dose compared with the reference DTB tablet [85]. Oral administration of a Su-SNEDDS of DTB to male SD rats resulted in a 2.7-fold increase in AUC and a 1.8-fold higher *C*_max_ compared with the DTB suspension [67]. Intestinal absorption of DTB nanoemulsions was evaluated using the in situ intestinal perfusion technique in rabbits, which demonstrated an approximately 3.3-fold reduction in the intestinal length required for drug absorption from the ileum, jejunum, duodenum, and colon compared with DTB solution [65]. DTB-loaded micelles incorporated into spray-dried microparticles, when orally administered to male Wistar rats, exhibited a 1.1-fold increase in AUC compared with the pure drug. This improvement was attributed to enhanced rate and extent of absorption [70]. The pharmacokinetics and intestinal uptake of mucoadhesive chitosan–lecithin hybrid nanoparticles were evaluated in female BALB/c mice following oral administration. Fluorescence imaging of the intestine demonstrated increased signal intensity, indicating strong adherence of the nanoparticles to the intestinal mucus layer, thereby facilitating enhanced absorption. The plasma concentration-time profile revealed a 5.08-fold increase in AUC and a 1.34-fold increase in *C*_max_ for the hybrid nanoparticles compared with free DTB. These findings suggest that the combined effects of mucoadhesion, nanoscale particle size, and chitosan-mediated permeation enhancement contribute to improved pharmacokinetic performance [36]. Polymeric micelles of DTB, when administered to rats, exhibited a lower initial plasma concentration but a prolonged systemic circulation time compared with the free drug. Notably, a 13-fold increase in *C*_max_ and a 2.8-fold increase in AUC were observed, indicating enhanced bioavailability along with increased plasma residence time [71]. For a summarization see Table 5.

**Table 5:** Summarization of the pharmacokinetic studies of different nanotechnology-enabled delivery systems.

Drug	Nanoformulation	Experimental model	Key pharmacokinetic outcomes	Relative bioavailability enhancement	Ref.

bosutinib	SLNs	male Wistar rats	increased MRT and plasma exposure	2.28-fold	[24]
bosutinib	folate-conjugated cubosomes	rat model	MRT = 15.35 h; increased AUC and *C*_max_	not numerically specified	[82]
bosutinib	lipid nanocarriers	SD rats	increased systemic exposure and reduced variability in fed and fasted states, lymphatic uptake is the primary mechanism	1.36–2.6-fold	[32]
bosutinib	biotin-functionalized liposomes	rat plasma study	*C*_max_ = 79.07 ± 5 µg/mL vs 4.7 ± 1 µg/mL (pure drug); markedly elevated AUC	≈38-fold AUC increase	[78]
nilotinib	amorphous nanosuspension	male SD rats	1.5-fold increase in *C*_max_ and 1.46-fold increase in AUC	1.46-fold	[48]
nilotinib	SMEDDS	rat model	approximately twofold increase in AUC	≈2-fold	[66]
nilotinib	yeast glucan particles	rat crossover study	doubled *C*_max_ and AUC; lymphatic *C*_max_ increased 4-fold	2.7-fold BA, 10-fold via lymph uptake	[93]
nilotinib	Tween 20-solubilised system	male SD rats model	markedly enhanced plasma exposure	5.2-fold	[92]
dasatinib	SLNs	male Wistar rats, two groups	improved oral absorption profile	2.28-fold	[20]
dasatinib	ASD	albino New Zealand white rabbits	*T*_max_, *C*_max_, and AUC increased by 4.3-, 2.0-, and 1.5-fold	145% increase	[62]
dasatinib	ASD (Dasynoc)	healthy male human subjects	2.3–4.8-fold increase in absorption	bioequivalent at 30% lower dose	[85]
dasatinib	polymeric micelles	male Wistar rats model, 2 groups (*n* = 6)	MRT prolonged 1.3-fold; *C*_max_ increased from 12.33 to 160.01 (μg/mL), no change in *T*_max_	2.8-fold AUC increase	[71]
dasatinib	lecithin-chitosan nanoparticle	BALB/c mice	1.34-fold increase in *C*_max_, increased retention in the intestinal mucosa	5.08-fold AUC increase	[36]
dasatinib	Su-SNEDDS	in vivo digestion model-male SD rats	13.5-fold higher aqueous concentration; 1.9-fold increase in *C*_max_, no change in *T*_max_	2.7-fold	[67]

### Regulatory and translational perspective

#### Quality by design

**Figure 5:**
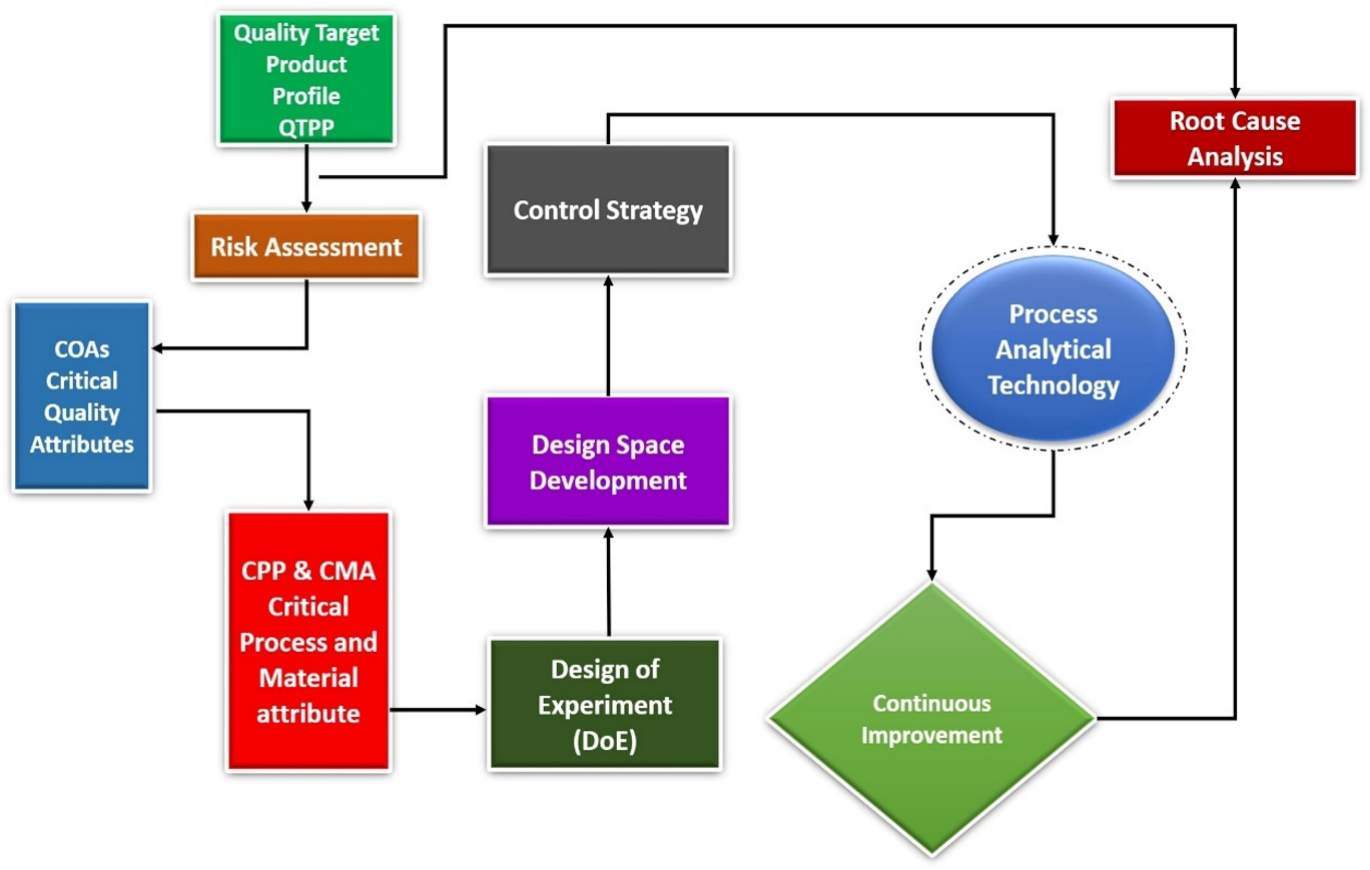
QbD framework for pharmaceutical product development.

The quality by design (QbD) framework, endorsed by the FDA and European Medicines Agency (EMA) through International Council for Harmonisation guidelines Q8–Q12, involves systematically identifying critical quality attributes (CQAs), critical process parameters (CPPs), and critical material attributes (CMAs) to understand the relationships between product and process variables. This framework is integrated with quality risk management strategies to ensure robust product development and consistent quality [94]. Although the QbD approach is increasingly being applied at the laboratory scale during formulation development, several nuances continue to hinder the scale-up of these formulations to commercial manufacturing. Consequently, establishing robust regulatory guidelines and obtaining regulatory approval remain challenging. Major obstacles include differences in quality control requirements, equipment limitations, and transport mechanisms compared with conventional dosage forms [95]. Building quality into the product from the initial stages of manufacturing through to the final stage (throughout the product lifecycle) helps to mitigate scale-up and regulatory challenges. The comprehensive workflow of the QbD approach, including risk assessment, design space development, process analytical technology, and control strategy, is illustrated in Figure 5.

**Figure 6:**
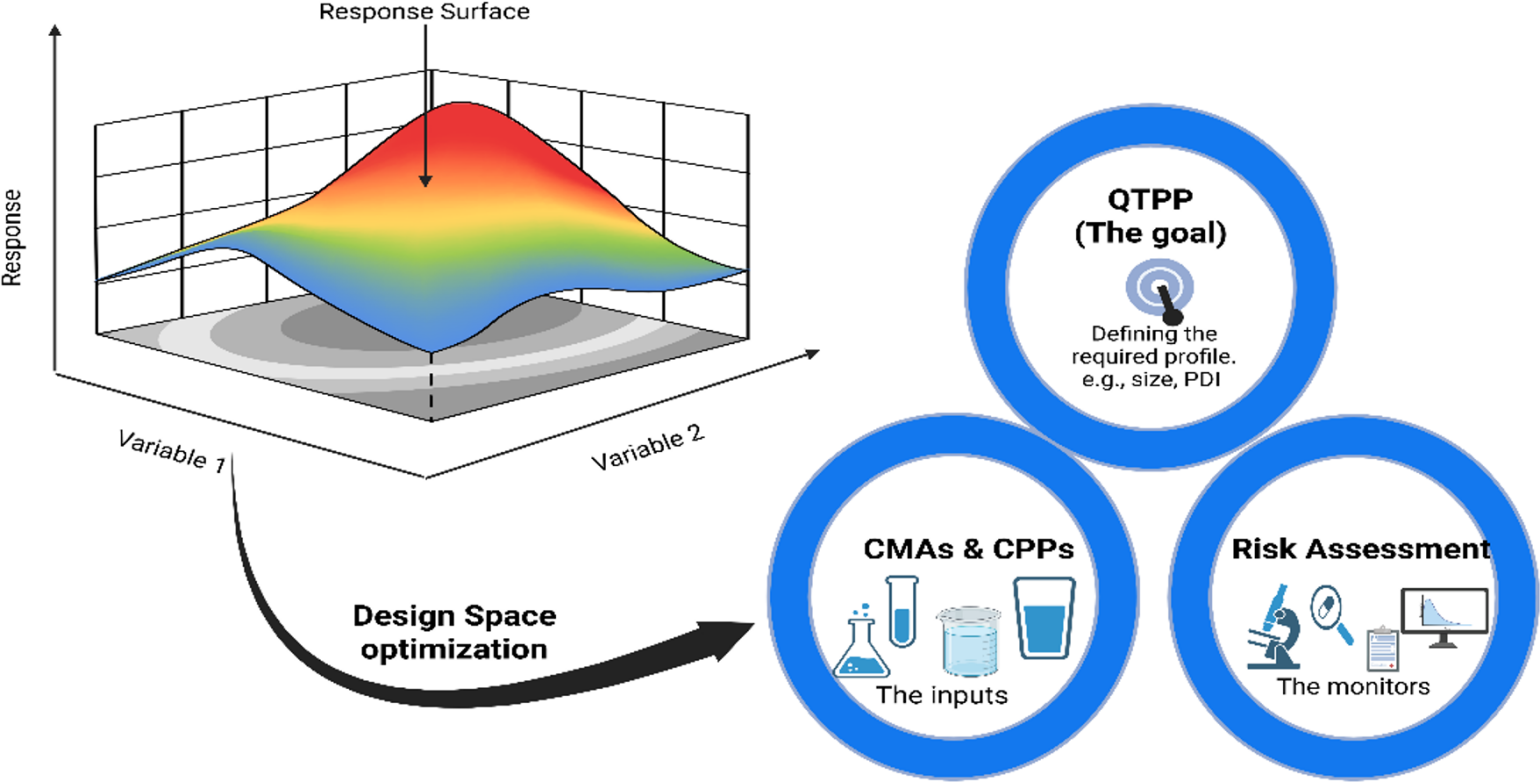
Design space optimisation via response surface methodology. Figure 6 was created in BioRender. Mishra, B. (2026) https://BioRender.com/t18aalv. This content is not subject to CC BY 4.0.

As illustrated in the flowchart in Figure 5, the first step is to define the quality target product profile (QTPP). For nanosystems, typical QTPP attributes include particle size below 500 nm, PDI below 0.2, uniform morphology, zeta potential of ±30 mV, encapsulation efficiency greater than 80%, and a desirable in vitro drug release profile. These parameters are generally considered dependent variables during experimental design to obtain an optimised formulation batch. In contrast, the independent variables include CPPs and CMAs that significantly influence nanosystem characteristics and, consequently, the bioavailability enhancement potential of the TKI delivery system.These variables are initially identified using screening designs, such as the Plackett–Burman design, which facilitates the selection of the most influential parameters from a large number of variables. The identified factors are subsequently used as independent variables in optimisation designs to define the design space [96]. Various designs of experiments approaches, employing different mathematical models and statistical tools, have been utilised to formulate TKI-based nanosystems. These include central composite design (CCD), Box–Behnken design (BBD), and full factorial designs using linear and quadratic models, along with statistical analyses such as ANOVA and *t*-tests. For instance, Panigrahi et al. employed a CCD-based optimisation strategy for BTB-loaded lipid nanoparticles. Particle size, PDI, EE, and zeta potential were selected as CQAs. The lipid and surfactant types for the SLN formulation were initially selected through preliminary screening, while pericrol concentration and poloxamer 188 percentage were identified as critical formulation factors influencing the CQAs. The optimisation study was conducted using a CCD with seven experimental runs [22]. Similarly, Famta et al. developed a BTB-loaded SLN formulation by CCD in which homogenization speed (rpm), drug content (% w/w), and surfactant concentration were identified as critical formulation variables among the various parameters influencing formulation development [32]. DTB- and hesperidin-loaded SLNs were also optimised using a CCD, with sonication time, poloxamer concentration, and camphor oil concentration selected as the independent variables [25]. Folate-functionalized BTB-loaded cubosomes were optimised using a CCD, in which lipid concentration, folate conjugation ratio, and processing temperature were selected as critical factors. The optimised formulation achieved a twofold enhancement in bioavailability [82]. A three-level, three-factor BBD comprising 17 experimental runs was employed to optimise DTB-loaded lecithin–chitosan mucoadhesive nanoparticles using a QbD approach. The lecithin-to-chitosan ratio, drug content, and surfactant concentration were selected as the independent variables, and the optimised formulation demonstrated a fivefold increase in bioavailability [36]. An I-optimal design employing linear, quadratic, and cubic models was selected to optimise DTB-loaded Su-SNEDDSs for enhanced bioavailability [67]. A similar optimisation approach was applied to DTB-loaded nanoemulsions, in which critical formulation factors, including oil percentage, surfactant concentration, and co-surfactant concentration, were systematically evaluated [86]. Full factorial designs, which involve more experimental runs, have also been employed by researchers, as they provide comprehensive information on the effects of individual factors and their interactions. Overall, the application of QbD-based experimental designs has enabled systematic identification and optimisation of critical formulation and process variables in TKI nanosystems. The use of statistical tools such as CCD, BBD, I-optimal, and full factorial designs has improved formulation robustness, reproducibility, and bioavailability outcomes. Collectively, these approaches facilitate rational scale-up and regulatory translation of nanotechnology-enabled TKI delivery systems. The relationship between formulation variables, design space optimisation, and critical quality attributes is further illustrated in Figure 6, highlighting the role of design of experiments in nanosystem development.

#### Scale-up challenges

**Figure 7:**
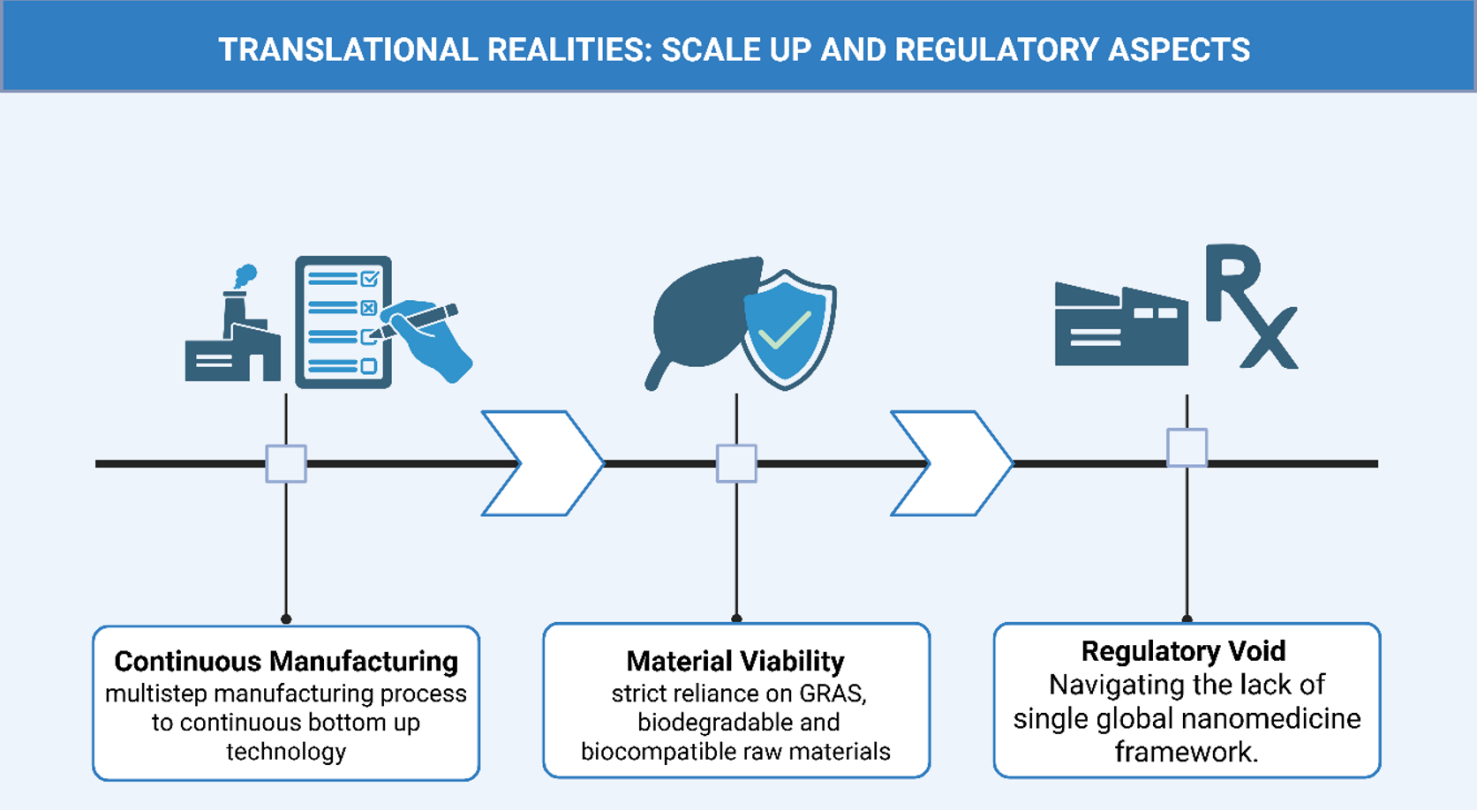
Translational and regulatory challenges in nanoformulation development. Figure 7 was created in BioRender. Mishra, B. (2026) https://BioRender.com/o7ygj7t. This content is not subject to CC BY 4.0.

Even though nanosystems developed to enhance the bioavailability of the second-generation TKIs provide several advantages over conventional dosage forms, the major challenge which ultimately decides if these dosage forms will reach the patients and give the desired results is scale-up to industrial level due to its complex multistep manufacturing process; they need to be manufactured in bulk quantity while maintaining the desired quality, purity, and stability after preclinical and clinical testing [97]. The lab-to-market transition of these nanoformulations is possible only when scale-up challenges are understood and addressed efficiently. The key translational and regulatory challenges associated with nanoformulation development are summarized in Figure 7. Beyond improved pharmacokinetic performance, the clinical viability of the nanoformulations for DTB, NTB, and BTB will be governed by post-formulation development potential, like regulatory strategy, chronic safety and toxicology, long-term physical stability, and manufacturing scale-up.

Paliwal et al. clearly describe the scale-up challenges, such as the selection of raw materials and production method [95]; they note that controlling particle size and shape while ensuring scalability and reproducibility is possible by employing continuous manufacturing via bottom-up technology, as reviewed in detail regarding various nanoparticle preparation methods by Costa and co-workers [98]. Thus, optimizing the production method and production parameters according to industrial requirements, employing QbD, and using generally regarded as safe, biodegradable, biocompatible, and nontoxic raw materials are prerequisites to limit scale-up challenges [95]. To summarize, at one extreme are the ASD and nanocrystals, whose manufacturing is continuous, good manufacturing practice (GMP)-validated, and supported by established process analytical technology; at the other extreme are the polymeric nanoparticles and liposomes, which require a specialized manufacturing facility for bulk production and come with significant process validation challenges.

#### Gaps regarding chronic toxicological/safety and long-term stability studies

Across the reviewed studies, the lack of safety and toxicology studies was the most prominent deficiency. Most preclinical studies reported only acute toxicology studies. Chronic myelogenous leukaemia patients receive TKI therapy for almost a lifetime; the safety of chronic oral exposure to novel nanoformulation excipients has not yet been established. For instance, in oral administration of PLGA nanoparticles, accumulation of microparticulate polymer in intestinal macrophages can activate potential inflammatory signalling, requiring further investigation. For surfactant-rich nanoformulations, cumulative hepatotoxic potential at therapeutic doses in cancer patients with concurrent TKI-related hepatic stress requires dedicated ICH S7A/S7B toxicological studies. Native β-cyclodextrin is nephrotoxic and can cause cholesterol depletion at chronic oral doses. Nanocrystals and ASDs are found to be safest, among all, in terms of acute and sub-acute toxicological studies. Moreover, ICH Q1A guidelines require 12-month real-time and 6-month accelerated stability data, both of which are missing.

#### Regulatory guidelines for nanomedicine

In the regulatory approval context, Zelboraf^®^ (ASD), Rapamune^®^ tablet (nanocrystal), Neoral^®^ (SMEDDS) [99] and Sporanox^®^ (CD complex) [100] as approved products for oral administration indicate that four out of eight evaluated platforms have the scope of reaching commercial approval. Regulatory approval of nanomedicines considers both drug substance and drug product attributes; however, greater emphasis is placed on nanoparticle-specific characteristics. Despite this, a well-defined global regulatory framework for the clinical translation of bioavailability-enhancing TKI-loaded nanosystems remains lacking, posing a significant challenge for manufacturers. Therefore, the application of advanced analytical and translational evaluation techniques to comprehensively assess the quality, safety, and efficacy of nanomedicines in humans is essential to meet regulatory requirements through robust documentation and reporting [101]. Major regulatory agencies, such as FDA and EMA, encourage the adoption of a QbD framework that emphasizes the identification and evaluation of critical quality attributes. This assessment extends beyond physicochemical characterization to include transport mechanisms, stability, pharmacokinetics, biodistribution, pharmacodynamics, and nanotoxicity, thereby encompassing both product quality and product safety evaluations [102]. The FDA has issued several guidance documents on nanomedicine development and regulatory authorization, available on its official website, that outline the requirements for approval of drug substances and drug products containing nanomaterials. The FDA’s 2019 guideline on lipid-based drug delivery systems and the 2022 guideline for drug products containing nanomaterials provide a primary regulatory framework [103]. Similar regulatory guidance frameworks have also been established by other agencies, including EMA and the Central Drugs Standard Control Organization (CDSCO). To evidence the clinical success of ASDs, Dasynoc^®^ (XS004) was the first second-generation TKI nanoformulation to receive orphan drug designation from the FDA and was launched for marketing by Xspray Pharma in September 2024. It is an amorphous solid dispersion of DTB that enhances bioavailability, reduces pharmacokinetic variability, and enables approximately a 30% reduction in the administered dose [104]. The same company submitted a new drug application for NTB hybrid amorphous solid nanoparticles in August 2025, reporting a dose reduction of approximately 50%, as stated in the company’s press release [105]. Other than ASDs, no nanoformulation of DTB, BTB, or NTB has completed a phase-II clinical trial, which may be due to poorly validated protocols for transitioning from animal to human studies for oral nanoformulation systems. Bioavailability enhancement in preclinical trials cannot be directly translated into humans; it will be an overprediction unless and until human clinical trials show similar bioavailability improvement. To conduct clinical trials to assess the safety, efficacy, and potency of the nanoformulation, a GMP-grade nanoformulation manufactured in GMP-compliant equipment/manufacturing facility at batch scales is a must. Most academic groups cannot access such facilities, representing a substantial and widening translational gap.

#### Future perspective and research recommendations

To improve the oral bioavailability of the BCS class-II/IV second-generation TKIs DTB, BTB and NTB considering the critical aspects of safety, stability, and scale-up for future translational potential, research could be performed regarding nanoplatforms incorporating excipients like TPGS for additional P-gp inhibition, polymers for solubility enhancement and stabilization, and materials like peptides, antibodies, or polymers for targeted delivery. A 12-month ICH-compliant stability study must be performed on promising nanoformulations. If the results are positive, phase-I clinical trials comparing bioavailability should be conducted to provide clinical evidence of the nanoformulation’s benefits. While preclinical results demonstrate improved bioavailability, translating them to clinical utility requires GMP manufacturing capability, dedicated investigational new drug toxicology studies, and crossover clinical bioavailability studies in patients, all achievable through industry–academia partnerships.

## Conclusion

The review comprehensively highlighted the role of nanosystem-based drug delivery strategies in addressing the critical solubility and permeability barriers associated with second-generation tyrosine kinase inhibitors. The critical analysis of published nanoformulation studies includes various true nanosystems of dasatinib, bosutinib, and nilotinib. The collective evidence demonstrates the potential benefits of each nanoformulation in markedly improving aqueous solubility, permeability, oral bioavailability, pharmacokinetic performance, and therapeutic efficacy. Furthermore, the successful integration of quality by design principles in the development of these nanosystems enables systematic optimization, improved reproducibility, and scalability, which are essential for clinical translation. Collectively, these advances support the evolution of robust, cost-effective, and patient-centric nanomedicines that hold significant promise for overcoming biopharmaceutical limitations of BCS class-IV TKIs and ultimately improving outcomes in cancer therapy. Upon critical analysis, it was found that a single nanoformulation platform is not universally superior to others regarding all criteria. But, when evaluated based on the relevant criteria (solubility enhancement, permeability/P-gp efflux bypass, safety profile, long-term physical stability, manufacturing scalability, and regulatory translation readiness), a single formulation can be selected, like nanocrystals or SNEDDSs; also, ASDs stand out among all other nanoformulations in terms of scalability. One of the major concerns is that no nanoformulation of dasatinib, bosutinib, and nilotinib has yet entered clinical trials; hence, a fundamental shift in research strategy from laboratory level optimization to industry level scale-up is required. The need for this is clear as variability in oral TKI exposure used for chronic treatment of cancer leads to dose-dependent side effects, that is, toxicity above therapeutic concentration, and treatment failure due to inadequate molecular response below therapeutic concentration. Preclinical data suggest that some nanoformulations can limit the inter- and intra-subject pharmacokinetic variability but clinically validated is only the product Dasynoc^®^; dedicated clinical pharmacokinetic trial data is required to extend this claim to other nanosystems. Nanosystems and nanotechnology-enabled delivery systems are novel delivery systems that can eliminate the food effects and pH-dependent solubility, as well as improve and maintain the mean oral bioavailability (by improving aqueous solubility and intestinal permeability). They have the potential to meaningfully improve the clinical outcomes and the quality of life of chronic myelogenous leukaemia patients, who require life-long anticancer therapy to prevent the progression of disease from the chronic phase to accelerated and blast phases (in the last phase, bone marrow transplant is the only treatment option to save the life of patient). To realize this potential, moving from promising preclinical results compiled in this review towards clinical study in humans with scientifically relevant approaches, after establishing manufacturing, stability, safety, and regulatory approval readiness is required.

## Data Availability

Data sharing not applicable to this article as no new datasets were generated or analysed in this review article.
